# Mean-field models for heterogeneous networks of two-dimensional integrate and fire neurons

**DOI:** 10.3389/fncom.2013.00184

**Published:** 2013-12-27

**Authors:** Wilten Nicola, Sue Ann Campbell

**Affiliations:** Department of Applied Mathematics, University of WaterlooWaterloo, ON, Canada

**Keywords:** integrate-and-fire neuron, mean-field model, hippocampus, bifurcation analysis, bursting

## Abstract

We analytically derive mean-field models for all-to-all coupled networks of heterogeneous, adapting, two-dimensional integrate and fire neurons. The class of models we consider includes the Izhikevich, adaptive exponential and quartic integrate and fire models. The heterogeneity in the parameters leads to different moment closure assumptions that can be made in the derivation of the mean-field model from the population density equation for the large network. Three different moment closure assumptions lead to three different mean-field systems. These systems can be used for distinct purposes such as bifurcation analysis of the large networks, prediction of steady state firing rate distributions, parameter estimation for actual neurons and faster exploration of the parameter space. We use the mean-field systems to analyze adaptation induced bursting under realistic sources of heterogeneity in multiple parameters. Our analysis demonstrates that the presence of heterogeneity causes the Hopf bifurcation associated with the emergence of bursting to change from sub-critical to super-critical. This is confirmed with numerical simulations of the full network for biologically reasonable parameter values. This change decreases the plausibility of adaptation being the cause of bursting in hippocampal area CA3, an area with a sizable population of heavily coupled, strongly adapting neurons.

## 1. Introduction

As computers become more powerful, there is a move to numerically simulate larger and larger model networks of neurons (Izhikevich and Edelman, 2008). While simulation is useful for confirming observed behavior it is not as helpful in determining the mechanisms underlying the behavior. The tools of dynamical systems theory, such as bifurcation analysis can be useful in this regard when studying single neuron or small network models. However, they are not viable for large networks, especially if the neurons are not identical. Thus, a common approach is to try to extrapolate large network behavior from detailed analysis of the behavior of individual cells or small networks (Skinner et al., 2005). This can be problematic as networks can have behavior that is not present in individual cells. For example, individual neurons that are only capable of tonic firing when isolated may burst when coupled in a network (van Vreeswijk and Hansel, 2001). Further, large networks may exhibit behavior not present in smaller networks. For example, Dur-e-Ahmad et al. studied bursting in networks ranging in size from 2 cells to 100. They found that bursting occurred in a larger range of parameters for larger networks (Dur-e-Ahmad et al., 2012, Figure 7).

Given the role of bursts in networks of neurons, it is important to understand how a network transitions (bifurcates) from a non-bursting behavior, to bursting. Bursting has been suggested to be a fairly important and information dense firing mode for neurons. For example, single bursts can induce long term potentiation and depression in the hippocampus, which are important processes for learning and memory (Lisman, 1997). Additionally, bursts have been found to carry more information about an animal's position in space than isolated spikes alone as place fields have been found to be more accurately defined when considering bursts alone (Lisman, 1997). Additionally, the mechanism we analyze in this paper, adaptation induced bursting has also been suggested as a biologically plausible mechanism for the generation of grid cells via oscillatory interference of the bursting (Zilli and Hasselmo, 2010). However, adaptation induced bursting is a network level phenomenon and we cannot apply bifurcation analysis directly to a network of adapting neurons.

Mean-field theory offers one approach to bridge this gap. In applying this theory, one usually derives (or suggests) a low dimensional system of differential equations that govern the moments of different variables across the network (Bressloff, 2012). For example, for a network of all-to-all coupled Izhikevich neurons (Izhikevich, 2003), one can derive a two dimensional system of differential equations for the mean adaptation variable and the mean synaptic gating variable (Nicola and Campbell, 2013). Mean-field systems enable one to conduct network level bifurcation analysis and hence to test different hypotheses about large network behavior. For example, Hemond et al. (2008) found that when uncoupled, the majority of hippocampal pyramidal neurons in region CA3 do not display bursting. This is contradictory to the observation that burst firing is ubiquitous in this region (Andersen et al., 2006, section 5.3.5). A possible explanation for these contradictory observations is that bursting is a network level phenomenon. This hypothesis has been tested using bifurcation analysis of the mean-field system derived from a network of Izhikevich neurons (Dur-e-Ahmad et al., 2012). In particular, it was shown that for a network of identical all-to-all coupled neurons fit to experimental data from Hemond et al. (2008) for CA3 pyramidal cells, bursting occurs for a large range of synaptic conductances and applied currents if the spike frequency adaptation in the neurons is sufficiently strong.

Hemond et al. (2008) also observed that the pyramidal neurons in their study were heterogeneous, in particular, the neurons had different degrees of spike frequency adaptation. When the study of Dur-e-Ahmad et al. (2012) was extended to a network of two homogeneous subpopulations of hippocampal neurons with different degrees of spike frequency adaptation, the mean-field equations predict, and numerical simulations confirm, that the region in the parameter space where bursting occurs decreases in size (Nicola and Campbell, 2013). This would seem to indicate that adaptation induced bursting may not be robust to heterogeneity, however, it is unknown how robust this network level bursting is to heterogeneity in different parameters. An extension of the mean-field system in Nicola and Campbell (2013) to large networks of heterogeneous neurons, is needed to fully analyze the robustness of adaptation induced bursting.

The application of mean-field analysis to large networks of heterogeneous neurons has been primarily limited to networks of one dimensional integrate and fire neurons with heterogeneity in the applied current (Hansel and Mato, 2001, 2003; Vladimirski et al., 2008). Hansel and Mato (2001, 2003) analyze a network of all-to-all coupled quadratic integrate and fire neurons consisting of two subpopulations: one excitatory and one inhibitory. They showed analytically that the tonic firing asynchronous state can lose stability either to a synchronous tonic firing state or a bursting state. Vladimirski et al. (2008) analyze a network of all-to-all coupled linear integrate and fire neurons subject to synaptic depression. This model was used to study network induced bursting in the developing chick spinal cord. Their derivation of the mean-field model is based on temporal averaging of the fast synaptic gating variable, in addition to the usual network averaging. This results in a model that only involves the distribution of slow synaptic depression variable and the distribution of firing rates. Vladimirski et al. note that, for their model, increased heterogeneity tends to make population induced bursting more robust. They also note that one cannot understand the behavior of their network with a single slow, network averaged synaptic depression variable.

More recently, Hermann and Touboul (2012) considered networks with heterogeneity in the synaptic conductances. They compare heterogeneity induced by a distribution of parameters and heterogeneity induced by noise. For a firing rate (Hopfield-type) model, they derive a mean field representation of the situation with noise, which is a system of two ODEs for the mean and variance of the network average voltage. They show that increasing the strength of the noise, (which corresponds to the variance of the heterogeneity) causes a transition from quiescence to oscillations in both the mean-field model and the full network simulations both with noise and a distribution of parameters. Based on these results, they suggest a mean field model for a network of excitable Fitzhugh Nagumo neurons, which consists of coupled ODEs for the network mean voltage and network mean recovery variable. In both the mean-field model and full network simulations they observe a transition from quiescence to periodicity and then to chaos as the variance of the heterogeneity is increased.

The motivation for the present paper is to explore the effect of heterogeneity in parameters on network induced bursting when adaptation is the primary negative feedback acting on the individual firing rates. To this end, we introduce a set of mean-field equations for networks of heterogeneous two-dimensional integrate and fire neurons. While the specific neural model we consider is for neurons with adaptation, our derivation is quite general and could be applied to other integrate and fire models. In contrast with (Vladimirski et al., 2008) our derivation of the mean-field model does not use temporal averaging thus we end up with a mean-field system which involves the network averaged synaptic gating variables as well as the distribution of adaptation variables. We also allow for heterogeneity in more than one parameter. Our approach is a generalization of that used for homogeneous networks of two dimensional integrate and fire neurons (Nicola and Campbell, 2013), however, in the heterogeneous case it turns out that there are actually multiple mean-field models, as different assumptions can be made during the derivation. This leads us to three distinct mean-field systems, each derived under different assumptions, and used for different purposes. Together, these sets of equations allow us to do bifurcation analysis on large networks, as in the homogeneous case. We show that the bifurcation structure of the heterogeneous network differs both qualitatively and quantitatively from the homogeneous network case. We discuss the implications of this for network induced bursting in the hippocampus.

When considering a homogeneous network, the mean-field variables are a good approximation for the variables of every neuron. However, this is not the case for a heterogeneous network. If the heterogeneity is large, then the neuron variables may be also widely distributed, rendering information about the first moments less useful. This also implies that the behavior of any individual neuron is less predictable with a mean-field system than it was in the homogeneous case. One of our mean-field systems addresses this problem, giving information about the distributions of the variables instead of just the mean.

When considering a model for a specific heterogeneous network of neurons, one stumbling block is determining the distribution of parameters. Estimates of the distribution can be made through direct intracellular recording of a sufficient number of neurons and conventional measurements of the biophysical properties (membrane capacitance, voltage threshold, etc.). Unfortunately, this is a very time consuming and intensive process. What is needed is a way of measuring the biophysical parameters of multiple neurons simultaneously. There are a few ways to sample multiple neurons such as multi-unit recordings using tetrode arrays, or two-photon microscopy techniques (Buzsáki, 2004; Grewe et al., 2010). However, these techniques typically only tell us about spike times of large (dozens to hundreds of neurons) networks (Buzsáki, 2004). While an impressive accomplishment, this still does not tell us anything directly about the biophysical properties of the neurons that caused those spikes. In this paper we use a mean-field system to determine an approximate distribution of firing rates for a network given a known distribution of parameters. This is an unusual state of affairs for a mean-field system, as these kinds of systems seldom give information about entire distributions. More importantly, however, using a mean-field system, we can invert a distribution of steady state firing rates (which can be obtained from multi-unit recordings) to obtain a distribution of parameters. In fact, this can be done at the individual neuron level, to determine the parameter value for any particular neuron. This allows one to estimate different biophysical parameters, which are difficult to measure at the network level, using easy to measure firing rate distributions. However, the assumptions required for the numerical accuracy of the estimation are fairly strong.

The plan for our article is as follows. Section 1.1 introduces the general class of adapting two-dimensional integrate and fire neurons used in our network models. This class was introduced by Touboul (2008), who also completed the bifurcation analysis of the single, uncoupled neuron. Population density methods are briefly introduced in section 1.2, as a population density equation serves as an intermediate step to obtain our mean-field models. Section 2 begins with a review of mean-field theory and the equations for the homogeneous network. This is followed, in sections 2.1–2.3, by the derivation of the three mean-field systems for the heterogeneous network. A comparison of numerical simulations of these mean-field systems and the full network is the subject of section 2.4. Applications of mean-field theory to networks with a single heterogeneous parameter can be found in section 3 including bifurcation analysis (section 3.1), distributions of parameters and firing rates (section 3.2) and using mean-field theory for parameter estimation from firing rate data (section 3.3). Applications of mean-field theory to networks with multiple sources of heterogeneity are included in section 4. A discussion of our work and its implications can be found in section 5.

## 2. Materials and methods

### 2.1. Non-linear integrate and fire neurons

We consider a network of two-dimensional integrate and fire models of the form

(1) $$\dot{v}=F(v)-w+I$$

(2) $$\dot{w}=a(bv-w),$$

where *v* represents the non-dimensionalized membrane potential and *w* serves as an adaptation variable. Time has also been non-dimensionalized. The dynamical equations (1, 2) are supplemented by the following discontinuities

(3) $$v\left(t_{\text{spike}}^{-}\right)=v_{\text{peak}}\Rightarrow \begin{array}{c} \begin{array}{l} v\left(t_{\text{spike}}^{+}\right)=v_{\text{reset}},\\ w\left(t_{\text{spike}}^{+}\right)=w\left(t_{\text{spike}}^{-}\right)+w_{\text{jump}}. \end{array} \end{array}$$

This particular notation was formally introduced by Touboul (2008), along with a full bifurcation analysis of this general family of adapting integrate and fire neurons. Members of this family include the Izhikevich model (Izhikevich, 2003), the adaptive exponential (AdEx) model (Brette and Gerstner, 2005; Naud et al., 2008) and Touboul's own quartic model (Touboul, 2008).

The methods of this paper can be applied to a network of any particular neuron belonging to this general family, and thus all derivations are done for this model. For the numerical examples, however, we only consider the Izhikevich neuron. In dimensional form this model is:

(4) $$C{\dot{V}}_{i}=k(V_{i}-V_{T})(V_{i}-V_{R})-W_{i}+I_{\text{app},i}$$

(5) $${\dot{W}}_{i}=\frac{\eta (V_{i}-V_{R})-W_{i}}{\tau_{W}}$$

(6) $$V_{i}(t_{\text{spike}}^{-})=V_{\text{peak}}\Rightarrow \begin{array}{c} \begin{array}{l} V_{i}\left(t_{\text{spike}}^{+}\right)=V_{\text{reset}}\\ W_{i}\left(t_{\text{spike}}^{+}\right)=W_{i}\left(t_{\text{spike}}^{-}\right)+W_{\text{jump},i}, \end{array} \end{array}$$

In dimensionless form, this model is given by Equations (1–3) with *F*(*v*) = *v*(*v* − α) in addition to dimensionless versions of the discontinuities (Equations 6). We will use uppercase letters for dimensional variables and lower case for their dimensionless counterparts. The application to other neural models is straight forward, see Nicola and Campbell (2013) where the homogeneous mean-field theory has been derived and tested for both the AdEx and the Izhikevich models.

Networks of these neurons can be coupled together through changes in the synaptic conductance. The synaptic conductance of post-synaptic neuron *i* due to presynaptic neurons *j* = 1, 2, …, *N* is given by

(7) $$g_{i}(t)=g_{i}s_{i}(t)=\frac{g_{i}}{N}\sum_{j=1}^{N}s_{ij}(t),$$

where *g*_*i*_ denotes the maximal synaptic conductance of neuron *i* and *s*_*i*_(*t*) denotes the total proportion of postsynaptic ion channels open in the membrane of neuron *i*. The time dependent variable *s*_*ij*_(*t*) represents the proportion of postsynaptic ion channels open in the membrane of neuron *i* as a result of the firing in neuron *j*.

The changes in *s*_*ij*_(*t*) that occur after a spike are often modeled as transient pulses. For example, if neuron *j* fires its *k*th action potential at time *t* = *t*_*j, k*_, then the variable *s*_*ij*_(*t*) at time *t* is given by

(8) $$s_{ij}(t)=\sum_{t_{j,k}<t}E(t-t_{j,k}).$$

There are different functions proposed for *E*(*t*) in the literature including the simple exponential, the alpha synapse and the double exponential. We primarily consider the simple exponential synapse

(9) $$E(t)=s_{\text{jump}}\exp\left(\frac{-t}{\tau_{s}}\right),$$

which is governed by the ordinary differential equation

(10) $$\frac{ds_{ij}(t)}{dt}=-\frac{s_{ij}}{\tau_{s}}+s_{\text{jump}}\sum_{t_{j,k}<t}\delta \left(t-t_{j,k}\right).$$

In the rest of the paper, we assume all-to-all connectivity and that the synaptic parameters *s*_jump_ and τ_*s*_ are the same for every synapse. In this case we may set *s*_*i*_(*t*) = *s*(*t*) for all *i*, as each postsynaptic neuron receives the same summed input from all the presynaptic neurons. Then, using Equations (7) and (10), the network of all-to-all coupled neurons that we consider is given by the following system of discontinuous ODE's:

(11) $${\dot{v}}_{i}=F(v_{i})-w_{i}+I_{i}+g_{i}s(t)(E_{r}-v_{i}),$$

(12) $${\dot{w}}_{i}=a_{i}(bv_{i}-w_{i}),$$

(13) $$\dot{s}=-\frac{s}{\tau_{s}}+\frac{s_{\text{jump}}}{N}\sum_{j=1}^{N}\sum_{t_{j,k}<t}\delta (t-t_{j,k}),$$

(14) $$v_{i}(t_{i,k}^{-})=v_{\text{peak}}\Rightarrow \begin{array}{c} \begin{array}{l} v_{i}(t_{i,k}^{+})=v_{\text{reset}},\\ w_{i}(t_{i,k}^{+})=w_{i}(t_{i,k}^{-})+w_{\text{jump}}. \end{array} \end{array}$$

for *i* = 1, 2, … *N*.

In the examples, we consider one or more parameters as the sources of heterogeneity. However, to simplify the notation in the derivations, we use the vector **β** to represent all the heterogeneous parameters. Then, denoting the state variables *v* and *w* as the vector ***x***, we can write the equations for the individual oscillator as

(15) $$\dot{x}=G(x,\beta ,s)=\left(\begin{array}{l} \begin{array}{l} G_{1}(x,\beta ,s)\\ G_{2}(x,\beta ) \end{array} \end{array}\right)$$

Given a specific heterogeneous parameter, *G*_1_ and *G*_2_ may not depend on **β**, or all of the components of **β**. However, for the sake of simplicity, we include the dependence in both equations.

Our numerical examples are restricted to the Izhikevich neural model, and we primarily consider the driving current *I*_*i*_ of each neuron, the synaptic conductance *g*_*i*_ and the adaptation jump size, *w*_jump, *i*_ as the source of heterogeneity. However, the mean-field equations we derive can be applied to any of the two-dimensional adapting integrate and fire models, with any heterogeneous parameter or set of parameters.

Finally, we note that in many applications *b* is a small parameter, and thus the *bv* term can be dropped in *G*_2_. We do this in all our numerical studies. However, one can still derive appropriate mean-field equations if this term is present (see discussion in Nicola and Campbell, 2013), and thus we have left the term in the derivations.

### 2.2. The population density equation

The population density function, ρ(***x**, t*) determines the density of neurons at a point in phase space, ***x***, at time *t*. Consider first the case of a homogeneous network, i.e., all the oscillators have the same parameter values, denoted by **β**. In the limit as *N* → ∞, one can derive the following evolution equation for the population density function:

(16) $$\frac{\partial \rho (x,t)}{\partial t}=-\nabla \cdot J(x,\beta ,s,t)$$

where **J** is given by

(17) $$J(x,\beta ,s,t)=G(x,\beta ,s)\rho (x,t)=\left(J^{V},J^{W}\right).$$

and must satisfy the boundary condition

(18) $$J^{V}(v_{\text{peak}},w,\beta ,s,t)=J^{V}(v_{\text{reset}},w+w_{\text{jump}},\beta ,s,t).$$

In the same limit, the differential equation for *s* converges to

(19) $$s^{\prime }=-\frac{s}{\tau_{s}}+s_{\text{jump}}\int_{W}J^{V}(v_{\text{peak}},w,s,\beta ,t)dw$$

where the integral term is actually the network averaged firing rate, which we denote as 〈*R*(*t*)〉. Derivations of Equation (16) can be found in various sources (Nykamp and Tranchina, 2000; Omurtag et al., 2000).

Equation (16) is frequently referred to as the continuity equation and it has various applications besides its use as an intermediate step in mean-field reductions. For example, the equation has been used to determine the stability of the asynchronous tonic firing state by various authors (Strogatz and Mirollo, 1991; Abbott and van Vreeswijk, 1993; van Vreeswijk et al., 1994; van Vreeswijk, 1996; Hansel and Mato, 2003; Sirovich et al., 2006). These papers predominantly consider homogeneous networks of linear integrate and fire neurons. The exception is the work of Hansel and Mato (2003) which considers heterogeneity in the applied current. One can study stability of various firing states using spectral analysis or other analytical treatments of this equation (Strogatz and Mirollo, 1991; Abbott and van Vreeswijk, 1993; van Vreeswijk, 1996; Knight, 2000; Sirovich et al., 2000, 2006; Hansel and Mato, 2003). However, these approaches are too complicated for the models we consider in this paper.

Now consider a heterogeneous network where the parameters vary from oscillator to oscillator, but are static in time. Then one can rewrite the equations for the individual oscillator as

(20) $${\dot{v}}_{i}=G_{1}(x_{i},\beta_{i},s),$$

(21) $${\dot{w}}_{i}=G_{2}(x_{i},\beta_{i}),$$

(22) $${\dot{\beta }}_{i}=0.$$

In this case the flux contribution due to **β** is 0 and the evolution equation for the network is given by

(23) $$\frac{\partial \rho (x,\beta ,t)}{\partial t}=-\nabla \cdot J(x,\beta ,s,t)$$

The density now has the vector of parameters, **β**, as an independent variable. The flux consists of the vector (*J*^*V*^, *J*^*W*^, 0), with **β** as an independent variable, as opposed to a fixed constant. If the parameters are time varying however, the final component of the flux will be non-zero. The equation for *s* is also different in the heterogeneous case:

(24) $$s^{\prime }=-\frac{s}{\tau_{s}}+s_{\text{jump}}\int_{W}\int_{\beta }J^{V}(v_{\text{peak}},w,s,\beta^{\prime },t)dwd\beta^{\prime }.$$

While the evolution equation (23) is an exact representation for the network in the large network limit, it is difficult to work with analytically. Additionally, as the dimensions of the PDE become large, it becomes difficult to find numerical solutions efficiently (Ly and Tranchina, 2007). However, mean-field reductions of the network can be used to reduce the population density PDE to a system of non-linear switching ODEs that governs the moments of the distribution. Unlike the PDE, the system of ODEs is tractable using bifurcation theory, at least numerically. Furthermore, we show that in the heterogeneous case, the resulting mean-field systems can yield more information than just the type of bifurcation that the network can undergo.

### 2.3. Mean-field theory

In the homogeneous case, the mean-field system of equations for an all-to-all coupled Izhikevich network was derived in Nicola and Campbell (2013). We present here a quick summary of this derivation. In order to derive a mean-field system of equations, one first needs to reduce the PDE for ρ(**x, t**) = ρ**(v, w, t)** by a dimension. This is done by first writing the density in its conditional form:

(25) $$\rho (v,w,t)=\rho_{V}(v,t)\rho_{W}(w|v,t)$$

and then integrating the continuity equation with respect to *w*. This yields the one dimensional PDE

(26) $$\frac{\partial \rho_{V}(v,t)}{\partial t}=-\frac{\partial G_{1}(v,\langle w|v\rangle ,s)\rho_{V}(v,t)}{\partial v}=-\frac{\partial J(v,\langle w|v\rangle ,s,t)}{\partial v},$$

where the flux has been redefined to

(27) $$J(v,\langle w|v\rangle ,s,t)=\int_{W}J^{V}(v,w,s,t)dw.$$

One can now make a first order moment closure assumption, 〈*w*|*v*〉 = 〈*w*〉, and derive an approximate ODE for 〈*w*〉, which yields the system

$$\begin{array}{l} \frac{\partial }{\partial t}\rho (v,t)=-\frac{\partial }{\partial v}\left((F(v)-\langle w\rangle +I+g(E_{r}-v)s)\left(\rho (v,t)\right)\right)\\ \;{\langle w\rangle }^{\prime }=\;\frac{b\langle v\rangle -\langle w\rangle }{\tau_{w}}+w_{\text{jump}}J(v_{\text{peak}},\langle w\rangle ,s,t)\\ \;s^{\prime }=-\frac{s}{\tau_{s}}+s_{\text{jump}}J(v_{\text{peak}},\langle w\rangle ,s,t) \end{array}$$

where the subscript on the density function has been dropped for convenience. The details and validity of the first order moment closure assumption that is used can be found in Ly and Tranchina (2007). We note, however, that the work in Ly and Tranchina (2007) was primarily with leaky integrate and fire neurons, as opposed to the two-dimensional adapting class we consider here. However, it is a necessary assumption to proceed analytically. If we assume that the adaptation time constant, $\tau_{w}=\frac{1}{a}$ is large, one can apply a quasi-steady state approximation to derive a system of switching ODE's for 〈*w*〉 and *s*:

(28) $${\langle w\rangle }^{\prime }=\frac{b\langle v\rangle -\langle w\rangle }{\tau_{w}}+w_{\text{jump}}\langle R_{i}(t)\rangle$$

(29) $$s^{\prime }=-\frac{s}{\tau_{s}}+s_{\text{jump}}\langle R_{i}(t)\rangle$$

(30) $$\langle R_{i}(t)\rangle =\{\begin{array}{ll} {\left[\int_{V}\frac{dv}{F(v)-\langle w\rangle +I+g(e_{r}-v)s}\right]}^{-1} & :H(\langle w\rangle ,s)\geq 0\\ 0 & :H(\langle w\rangle ,s)<0 \end{array}$$

The switching manifold for the system, *H*(〈*w*〉, *s*) is given by:

(31) $$H(\langle w\rangle ,s)=I-\langle w\rangle +\underset{v}{\min}(F(v)+g(e_{r}-v)s).$$

Note that *H*(〈*w*〉, *s*) depends on the parameter(s) of the model, and thus for the heterogeneous case, we make this dependence explicit by writing *H*(〈*w*〉, *s*, **β**). As the computation for 〈*v*〉 is somewhat lengthy and is only outlined in the discussion of Nicola and Campbell (2013), we have placed it in Appendix A. Note that this approach is similar to temporal averaging of a fast voltage equation assuming slow synaptic and adaptation currents, as outlined in Ermentrout (1998) and Ermentrout and Terman (2010).

For the Izhikevich neuron, Equations (30, 31) become

(32) $$\langle R_{i}(t)\rangle =\{\begin{array}{ll} \text{​​}{\left[\int_{V}\frac{dv}{v(v-\alpha )-\langle w\rangle +I+g(e_{r}-v)s}\right]}^{-1} & :H(\langle w\rangle ,s)\geq 0\text{​}\\ 0 & \text{​}:H(\langle w\rangle ,s)<0 \end{array}$$

(33) $$H(\langle w\rangle ,s)=I-\langle w\rangle -\frac{{\left(\alpha +gs\right)}^{2}}{4}+ge_{r}s.$$

Note that in this case, we can evaluate 〈*R*_*i*_(*t*)〉 explicitly:

$$\langle R_{i}(t)\rangle \text{​}=\text{​}\{\begin{array}{ll} \frac{\sqrt{I-I^{*}(\langle w\rangle ,s)}}{\arctan\left(\frac{v_{\text{peak}}-\frac{\alpha +gs}{2}}{\sqrt{I-I^{*}(\langle w\rangle ,s)}}\right)-\arctan\left(\frac{v_{\text{reset}}-\frac{\alpha +gs}{2}}{\sqrt{I-I^{*}(\langle w\rangle ,s)}}\right)} & :H(\langle w\rangle ,s)\geq 0\\ 0 & :H(\langle w\rangle ,s)<0 \end{array}$$

in addition to an approximation to 〈*v*〉

(34) $$\langle v\rangle =\{\begin{array}{ll} \frac{\langle R_{i}(t)\rangle }{2}\log\left(\frac{{\left(v_{\text{peak}}-\frac{\alpha +gs}{2}\right)}^{2}+H(\langle w\rangle ,s)}{{\left(v_{\text{reset}}-\frac{\alpha +gs}{2}\right)}^{2}+H(\langle w\rangle ,s)}\right)+\frac{\alpha +gs}{2} & :H(\langle w\rangle ,s)\geq 0\\ \frac{\alpha +gs}{2}-\sqrt{-H(\langle w\rangle ,s)} & :H(\langle w\rangle ,s)<0 \end{array}$$

**Figure 1:**
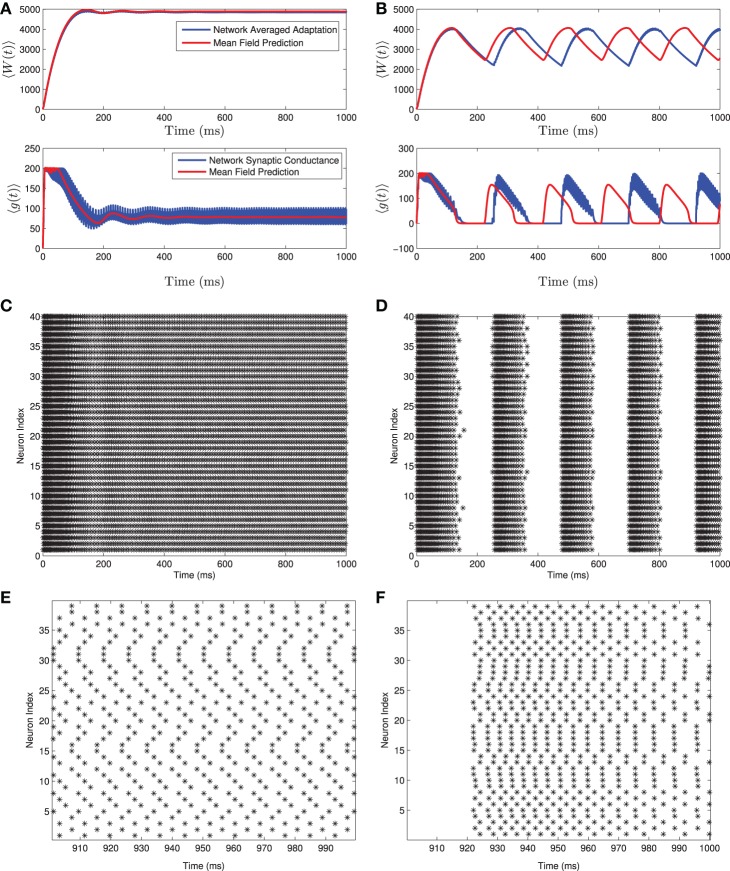
**Numerical simulation of a homogeneous network of 1000 Izhikevich neurons, with parameters as follows. (A,C,E)** *I*_app_ = 4500 pA, *g*_syn_ = 200 nS **(B,D,F)** *I*_app_ = 3500 pA, *g*_syn_ = 200 nS. The rest of the parameters can be found in Table 1. Simulations of the mean-field equations (in red) and the mean values of the corresponding full network simulations (in blue) showing **(A)** tonic firing and **(B)** bursting. In this and the following figures 〈*W*(*t*)〉 is the network mean adaptation variable in dimensional form and 〈*g*(*t*)〉 = *g*_syn_*s*(*t*) is the network mean synaptic conductance. **(B,D)** Raster plots for 40 randomly selected neurons from the network simulation in **(A,C)**. **(E,F)** are the last 100 ms of the raster plots in **(C,D)**, respectively. The mean-field equations are fairly accurate both when the network is tonically firing and when it is bursting. For the tonic firing case the neurons fire asynchronously at steady state, while in the bursting case, the neurons seem to align into two synchronously firing subpopulations firing out of phase with one another during the burst.

A comparison of solutions of these equations and the full network are shown in Figure 1 for both the tonic firing and the bursting case. Note that during tonic firing, the steady state firing of the individual neurons appears to be asynchronous, while during bursts the neurons separate into two synchronous subpopulations that fire out of phase with one another (see Figures 1E,F). While the mean-field system is more accurate when the neurons all fire asynchronously, the synchronization in the bursting state does not appear to be a substantial source of error in determining the mean-values and the resulting qualitative behaviors. The asynchronous firing in the non-bursting region is consistent with previous work on the stability of asynchronous states with excitatory coupled class one oscillators (Abbott and van Vreeswijk, 1993).

This system of equations is valid when τ_*w*_ » *O*(1), however, the magnitude of τ_*s*_ is also significant. While in the original derivation of Nicola and Campbell (2013), τ_*s*_ = *O*(τ_*w*_) was suggested as a criterion for validity, this is not actually necessary. One merely requires that τ_*s*_ not be significantly smaller than *O*(1), the time scale of the PDE. The reason for this is that if the time scale of the ODE for *s* is smaller than that of the PDE then the quasi-steady state approximation must be applied to the ODE for *s* as well. The requirements on the time constants are carried forward in the heterogeneous case.

In our models, the timescale of the ODE for *s* is typically between that of the PDE and that of the ODE for *w*, thus we have not applied the quasi-steady approximation to *s*. Applying a quasi-steady state approximation to both *s* and the reduced PDE yields a more compact system, which is just an ODE for 〈*w*〉, however, the analysis does not get any simpler. The reason for this is two-fold: the ODE for 〈*w*〉 remains non-smooth and the firing rate now has to be implicitly solved at each time step. Thus, it is more convenient to apply the quasi-steady state approximation only to the partial differential equation.

When parameter heterogeneity is added into the mix, it turns out that there are multiple “mean-field” systems of equations that can be derived, by applying different assumptions on the conditional moments. We outline three different assumptions that can be made and derive the resulting system of mean-field equations in each case.

#### 2.3.1. Mean-field system I

We begin by writing out the density function in the conditional form

(35) $$\rho (x,\beta ,t)=\rho_{x}(x,t)\rho_{\beta }(\beta |x,t)$$

The continuity equation is then given by

(36) $$\frac{\partial \left(\rho_{x}(x,t)\rho_{\beta }(\beta |x,t)\right)}{\partial t}=-\nabla \cdot J(x,s,\beta ,t).$$

Simple integration with respect to **β** yields the reduced continuity equation

(37) $$\frac{\partial \rho_{x}(x,t)}{\partial t}=-\nabla \cdot J(x,s,\langle \beta |x\rangle ,t).$$

This step is valid for all the non-dimensionalized models we consider as they are all linear in their dimensionless parameters (see Touboul, 2008). The flux has also been redefined upon integration to

$$(J^{V},J^{W})=\rho_{x}(x,t)\left(G_{1}(x,\langle \beta |x\rangle ,s),G_{2}(x,\langle \beta |x\rangle )\right).$$

We now apply the moment closure assumption 〈**β**|***x***〉 = 〈**β**〉 to yield the following PDE:

(38) $$\frac{\partial \rho_{x}(x,t)}{\partial t}=-\nabla \cdot J(x,s,\langle \beta \rangle ,t).$$

It should be clear that this is equivalent to the continuity equation for a homogeneous network with parameter values fixed at 〈**β**〉. Thus, the associated mean-field system is identical to the homogeneous case, only with the parameters fixed at 〈**β**〉. This is the simplest assumption one can make in the heterogeneous case. For example, if we treat *I* as the source of heterogeneity for a network of Izhikevich neurons, with distribution ρ_*I*_(*I*), then the resulting mean-field system is

(39) $${\langle w\rangle }^{\prime }=\frac{b\langle v\rangle -\langle w\rangle }{\tau_{w}}+w_{\text{jump}}\langle R_{i}(t)\rangle$$

(40) $$s^{\prime }=-\frac{s}{\tau_{s}}+s_{\text{jump}}\langle R_{i}(t)\rangle$$

(41) $$\langle R_{i}(t)\rangle =\{\begin{array}{ll} {\left(\int_{V}\frac{dv}{v(v-\alpha )-\langle w\rangle +\langle I\rangle +g(e_{r}-v)s}\right)}^{-1} & :H(\langle w\rangle ,s,\langle I\rangle )\geq 0\\ 0 & :H(\langle w\rangle ,s,\langle I\rangle )<0 \end{array}$$

(42) $$H(\langle w\rangle ,s,\langle I\rangle )=\langle I\rangle -\langle w\rangle -\frac{{\left(\alpha +gs\right)}^{2}}{4}+ge_{r}s$$

(43) $$\langle v\rangle =\{\begin{array}{l} \begin{array}{l} \frac{\langle R_{i}(t)\rangle }{2}\log\left(\frac{{\left(v_{\text{peak}}-\frac{\alpha +gs}{2}\right)}^{2}+H(\langle w\rangle ,s,\langle I\rangle )}{{\left(v_{\text{reset}}-\frac{\alpha +gs}{2}\right)}^{2}+H(\langle w\rangle ,s,\langle I\rangle )}\right)+\frac{\alpha +gs}{2}\\ \;:H(\langle w\rangle ,s,\langle I\rangle )\geq 0 \end{array}\\ \begin{array}{l} \frac{\alpha +gs}{2}-\sqrt{-H(\langle w\rangle ,s,\langle I\rangle )}\\ \;:H(\langle w\rangle ,s,\langle I\rangle )<0 \end{array} \end{array}$$

Note that *I* in Equations (32, 33) has been replaced by 〈*I*〉 in Equations (41–43). We treat this system as the baseline mean-field model for comparison purposes, in addition to direct numerical simulations of the network. We denote this system of equations as mean-field one (MFI). We should expect this system to be an adequate approximation to the actual network for narrowly centered distributions of the parameter heterogeneity (small values of the variance, σ_β_).

This set of differential equations is representative of a common approach taken when fitting actual neurons. In this approach, multiple estimates of parameters or measurements taken from multiple neurons are averaged to yield a single parameter value, which is really the mean parameter value, 〈**β**〉. Simulations of homogeneous, large networks are then run with the parameters fixed at their mean values. As we shall see in subsequent sections, the behavior of a simulated heterogeneous network can differ substantially from that of MFI.

#### 2.3.2. Mean-field system II

To derived our second mean-field system, we begin by writing the density function in the alternative conditional form

(44) $$\rho (v,w,\beta ,t)=\rho_{W}(w,t|\beta ,v)\rho_{V}(v,t|\beta )\rho_{\beta }(\beta ).$$

Next we integrate the continuity equation with respect to *w*. This yields the following system

(45) $$\begin{array}{l} \frac{\partial \rho_{V}(v,t|\beta )}{\partial t}\rho_{\beta }(\beta )=-\int_{W}\left(\text{​}\frac{\partial J^{V}(v,w,s,\beta ,t)}{\partial v}+\frac{\partial J^{W}(v,w,s,\beta ,t)}{\partial w}\text{​}\right)dw\\ \;=-\frac{\partial }{\partial v}J(v,\langle w|v,\beta \rangle ,s,\beta ,t)-J^{W}(v,w,s,\beta ,t){|}_{\partial W}\\ \;=-\frac{\partial }{\partial v}J(v,\langle w|v,\beta \rangle ,s,\beta ,t), \end{array}$$

where the last term vanishes as *J*^*W*^ is assumed to be vanishing on the boundary, and

(46) $$J(v,\langle w|v,\beta \rangle ,s,\beta ,t)=\int_{W}J^{V}(v,w,s,\beta ,t)dw.$$

We now make the first order moment closure assumption 〈*w*|*v*, **β**〉 = 〈*w*〉. Then to complete the system, we must derive a differential equation for 〈*w*〉:

(47) $$\begin{array}{l} {\langle w\rangle }^{\prime }=\int_{V}\int_{W}\int_{\beta }w\frac{\partial \rho (v,w,\beta ,t)}{\partial t}d\beta dwdv\\ \;=-\int_{V}\int_{W}\int_{\beta }w\left(\frac{\partial J^{W}}{\partial w}+\frac{\partial J^{V}}{\partial v}\right)d\beta dwdv\\ \;=\int_{V}\int_{W}\int_{\beta }G_{2}(v,w,\beta )\rho (v,w,\beta ,t)d\beta dwdv\\ \;-\int_{W}\int_{\beta }w(J^{V}(v_{\text{peak}},w,s,\beta ,t)-J^{V}(v_{\text{reset}},w,s,\beta ,t))d\beta dw\\ \;=\langle G_{2}(v,w,\beta )\rangle -\int_{W}\int_{\beta }w(J^{V}(v_{\text{peak}},w,s,\beta ,t)\\ \;-J^{V}(v_{\text{peak}},w-w_{\text{jump}},s,\beta ,t))d\beta dw \end{array}$$

(48) $$\begin{array}{l} =\langle G_{2}(v,w,\beta )\rangle +\int_{\beta }\int_{W}w_{\text{jump}}J^{V}(v_{\text{peak}},w,s,\beta ,t)dwd\beta \\ \;+\;O(w_{\text{jump}}^{2})\\ \approx G_{2}(\langle v\rangle ,\langle w\rangle ,\langle \beta \rangle )+\int_{\beta }w_{\text{jump}}J(v_{\text{peak}},\langle w\rangle ,s,\beta ,t)d\beta . \end{array}$$

Note that we have made the approximation 〈*G*_2_(*v, w*, **β**)〉 = *G*_2_(〈*v*〉, 〈*w*〉, 〈**β**〉) in addition to dropping the *O*(*w*^2^_jump_) terms. Additionally, the substitution in Equation (47) comes from the boundary condition (Equation 18).

Applying a quasi-steady state approximation to the PDE (Equation 45) yields the following equation for the steady state voltage independent flux, *J*(〈*w*〉, *s*, **β**):

(49) $$J(\langle w\rangle ,s,\beta )=\{\begin{array}{ll} {\left[\int_{V}\frac{dv}{G_{1}(v,\langle w\rangle ,s,\beta )}\right]}^{-1}\rho_{\beta }(\beta ) & \text{if }H(\langle w\rangle ,s,\beta )\geq 0\\ 0 & \text{if }H(\langle w\rangle ,s,\beta )<0 \end{array}.$$

We interpret the ratio *J*(〈*w*〉, *s*, **β**)/ρ_**β**_(**β**) as the parameter dependent (or conditional) network averaged firing rate, 〈*R*_*i*_(*t*)|**β**〉, based on the fact that

$$\int_{\beta }J(\langle w\rangle ,s,\beta )d\beta \approx \langle R_{i}(t)\rangle .$$

In other words, the distribution of parameters induces a distribution of firing rates across the network, and the network averaged firing rate is the mean of the distribution.

In summary, the resulting mean-field equations are given by:

(50) $${\langle w\rangle }^{\prime }=\frac{b\langle v\rangle -\langle w\rangle }{\tau_{w}}+\int_{\beta }w_{\text{jump}}\langle R_{i}(t)|\beta \rangle \rho_{\beta }(\beta )d\beta$$

(51) $$s^{\prime }=-\frac{s}{\tau_{s}}+s_{\text{jump}}\int_{\beta }\langle R_{i}(t)|\beta \rangle \rho_{\beta }(\beta )d\beta$$

(52) $$\langle R_{i}(t)|\beta \rangle =\{\begin{array}{ll} {\left[\int_{V}\frac{dv}{G_{1}(v,\langle w\rangle ,s,\beta )}\right]}^{-1} & :H(\langle w\rangle ,s,\beta )\geq 0\\ 0 & :H(\langle w\rangle ,s,\beta )<0 \end{array}$$

(53) $$H(\langle w\rangle ,s,\beta )=I-\langle w\rangle +\underset{v}{\min}(F(v)+g(e_{r}-v)s)$$

(54) $$\langle v\rangle =\int_{\beta }\langle v|\beta \rangle \rho_{\beta }(\beta )d\beta$$

where the forms of *G*_1_(*v*, 〈*w*〉, *s*, **β**) and *H*(〈*w*〉, *s*, **β**) depend on which specific neural model is used, and the equation for 〈*v*|**β**〉 can be found in Appendix A. Note that the distribution of firing rates is not computed explicitly in these equations, only the conditional firing rates, 〈*R*_*i*_(*t*)|**β**〉, are computed. However, we show in section 3.2.2 that a distribution for the steady state firing rates of the network can be computed using 〈*R*_*i*_(*t*)|**β**〉. We refer to Equations (50–54) as mean-field two (MFII).

It appears that MFII adds some smoothness to the non-smooth MFI equations. This can be easily seen by taking a heterogeneous background current to each neuron, *I*_*i*_. In this situation, each neuron has a total input current given by *I*_*i*_ + *I*_syn_, where *I*_syn_ is the synaptic current given by the network coupling. It follows that the network averaged firing rate is approximately a function of *I*_syn_

$$\int_{\beta }\langle R_{i}(t)|I+I_{\text{syn}}\rangle \rho_{I}(I)dI\approx F(I_{\text{syn}})$$

**Figure 2:**
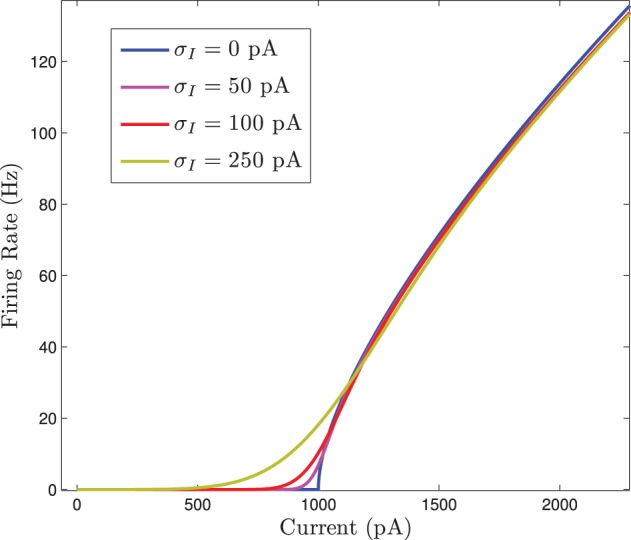
**The firing rate curve *F*(*I*) for a Gaussian distributed background current in the 〈*R*_*i*_(*t*)〉 response curve for the homogeneous network**. The *F*(*I*) curves are plotted for increasing values of σ_*I*_ which smooths out the square root type non-smoothness at the onset of firing.

If *I*_syn_ is treated as parameter, this equation can be evaluated with differing standard deviations for the normally distributed input current. When this is done we see that the *F*(*I*) curve becomes smoothed out as the standard deviation increases. This is shown in Figure 2.

Additionally, MFI and MFII also differ in the order in which the integrations are carried out. In MFI, we integrate with respect to **β** first, and then apply the first order moment closure assumptions 〈**β**|***x***〉 = 〈**β**〉 and 〈*w*|*v*〉 = 〈*w*〉. In MFII, we integrate with respect to *w* first, and then apply the moment closure assumption 〈*w*|*v*, **β**〉 = 〈*w*〉. Furthermore, if 〈*R*_*i*_(*t*)|**β**〉 does not actually depend on the heterogeneous parameter **β**, such as when the heterogeneity is in *w*_jump_, then MFI and MFII are identical.

The first order moment closure assumption used here can be weakened. This leads to the “mean-field” system in the next subsection, which is a different kind of system than MFI and MFII.

#### 2.3.3. Mean-field system III

Suppose that instead of assuming that 〈*w*|*v*, **β**〉 = 〈*w*〉, we make the weaker assumption that 〈*w*|*v*, **β**〉 = 〈*w*|**β**〉. It turns out that this assumption yields a PDE, even when one makes the quasi-steady state approximation, as we now show. Applying this weaker moment closure assumption to Equations (45) yields the following simplification of the continuity equation:

(55) $$\frac{\partial \rho_{V}(v,t|\beta )}{\partial t}\rho_{\beta }(\beta )=-\frac{\partial }{\partial v}J(v,\langle w|\beta \rangle ,s,\beta ,t).$$

Application of the quasi-steady state approximation now yields

$$\begin{array}{l} J(v,\langle w|\beta \rangle ,s,\beta )=\{\begin{array}{ll} {\left[\int_{V}\frac{dv}{G_{1}(v,\langle w|\beta \rangle ,s,\beta )}\right]}^{-1}\rho_{\beta }(\beta ) & :H(\langle w|\beta \rangle ,s,\beta )\geq 0\\ 0 & :H(\langle w|\beta \rangle ,s,\beta )<0 \end{array},\\ \;H(\langle w|\beta \rangle ,s,\beta )=I-\langle w|\beta \rangle +\underset{v}{\min}(F(v)+g(e_{r}-v)s). \end{array}$$

An equation for the time variation of 〈*w*|**β**〉 can be derived in a similar manner to the last section, yielding the following mean-field system:

(56) $$\frac{\partial \langle w|\beta \rangle }{\partial t}=\frac{b\langle v|\beta \rangle -\langle w|\beta \rangle }{\tau_{w}}+w_{\text{jump}}\langle R_{i}(t)|\beta \rangle$$

(57) $$\frac{ds}{dt}=-\frac{s}{\tau_{s}}+s_{\text{jump}}\int_{\beta }\langle R_{i}(t)|\beta \rangle \rho_{\beta }(\beta )d\beta$$

(58) $$\langle R_{i}(t)|\beta \rangle =\{\begin{array}{ll} {\left[\int_{V}\frac{dv}{G_{1}(v,\langle w|\beta \rangle ,s,\beta )}\right]}^{-1} & :H(\langle w|\beta \rangle ,s,\beta )\geq 0\\ 0 & :H(\langle w|\beta \rangle ,s,\beta )<0 \end{array}$$

Note that 〈*w*〉 can be computed via:

(59) $$\langle w\rangle =\int_{\beta }\langle w|\beta \rangle \rho_{\beta }(\beta )d\beta .$$

The equation for 〈*v*|**β**〉 can be found in Appendix A. We denote this system as mean-field three (MFIII). Note that the equation for 〈*w*|**β**〉 is actually a PDE. This is due to the fact that the conditional moments, 〈*w*|**β**〉, 〈*R*|**β**〉 and 〈*v*|**β**〉 are functions of both time and the “spatial” variable **β**. This partial differential equation is easier to deal with than most PDEs as it has no spatial derivatives, however, the right hand side of the differential equation is non-smooth.

While this system should be more accurate than mean-field II, it has the drawback of being more difficult to analyze. The dependence on **β** forces one to discretize over a mesh in **β** in order to work numerically with this system. This approach, typically referred to as the method of lines in the literature, is often used to solve PDE's with no spatial derivatives. We use this approach to numerically simulate Equation (56). In order to compute the integrals in Equations (57–59) using the method of lines, we choose a grid that is non-uniform and generated with the density function ρ_β_(**β**). The integrals are subsequently replaced with averaging over the entire grid, which is precisely a Monte–Carlo method for estimating the integrals.

Numerical bifurcation analysis of MFIII is more difficult as it is a PDE. In principle it is possible to do numerical bifurcation analysis on a PDE by discretizing and analyzing the resulting large system of coupled ODE's (Ko and Ermentrout, 2007). However, when we tried this approach on MFIII it proved to be too numerically intensive, and required a lengthy period of time for convergence of the numerical methods used for continuation. Additionally, the system of ODE's is still non-smooth, which causes problems with most numerical continuation software. However, as we shall show later, an approach that yields similar information to direct bifurcation analysis can be used with MFIII.

## 3. Results

### 3.1. Numerical simulations

We carried out numerical simulations of the full network model with a large number of neurons and the corresponding mean field systems. The large network simulations are carried out on the dimensional version of the equations. The results are presented in terms of the network mean adaptation, 〈*W*(*t*)〉, which is the dimensional version of 〈*w*〉, the network mean synaptic conductance, 〈*g*(*t*)〉 = *g*_syn_*s*(*t*), and the dimensional parameters described in Table 1. Since the mean field systems are given in terms of the dimensionless variables and parameters, results from mean field simulations are converted to dimensional form for comparison with the full network simulations.

**Table 1:** **The values of the model parameters and variances of the distributions used in this paper**.

**Dimensional parameters**		**Dimensionless parameters**	
*C*	250 pF		
*k*	2.5 nS/mV		
*V*_*R*_	−65 mV		
*V*_*T*_	$V_{R}+40-\frac{b}{k}$	$\alpha =1+\frac{V_{T}}{\left|V_{R}\right|}$	0.6215
	= 41.7 mV	$\alpha =1+\frac{V_{T}}{\left|V_{R}\right|}$	0.6215
*V*_peak_	30 mV	$v_{\text{peak}}=1+\frac{V_{\text{peak}}}{\left|V_{R}\right|}$	1.461
*V*_reset_	−55 mV	$v_{\text{reset}}=1+\frac{V_{\text{reset}}}{\left|V_{R}\right|}$	0.1538
*W*_jump_	200 pA	$w_{\text{jump}}=\frac{W_{\text{jump}}}{k|V_{R}{|}^{2}}$	0.0189
τ_*W*_	200 *ms*	$a={\left(\frac{\tau_{W}k|V_{R}|}{C}\right)}^{-1}$	0.0077
η	−1 nS	$b=\frac{\eta }{k|V_{R}|}$	−0.0062
*I*_app_	1000–5000 pA	$I=\frac{I_{\text{app}}}{k|V_{R}{|}^{2}}$	0.0776–0.3333
*g*_syn_	0–600 nS	$g=\frac{g_{\text{syn}}}{k|V_{R}|}$	0–3.6923
τ_syn_	4 ms	$\tau_{s}=\frac{\tau_{\text{syn}}k|V_{R}|}{C}$	2.6
*s*_jump_	0.8		
*N*	1000		
σ_*I*_	0–1000 pA		
σ_*g*_	50 nS		
σ_*d*_	50 pA		
*m* (mixing parameter)	0–1		

*These values apply unless otherwise indicated. Rheobase for the dimensional parameter values is *I*_rh_ = 1000 pA*.

Recall that simulations of a homogeneous network and the corresponding mean-field system are shown in Figure 1. Note that the network undergoes a bifurcation from tonically firing to bursting as the amount of applied current *I*_app_ is decreased, with all other parameter values held fixed. Further simulations show that if *I*_app_ is decreased below *I*_rh_ then all neurons in the network are quiescent (non-firing).

To determine and compare the validity of the three mean-field systems we derived for the heterogeneous networks, we have run a series of numerical simulations of these systems and of an actual network containing 1000 neurons. The parameter values for the individual neurons can be found in Table 1. They are based on those given in Dur-e-Ahmad et al. (2012) which were fit to data for hippocampal CA3 pyramidal neurons from Hemond et al. (2008). These are the parameter values we use for the rest of this paper, unless otherwise indicated.

As a starting point, we consider heterogeneity only in the applied current. The distributions are assumed to be normal with mean 〈*I*〉 and standard deviation σ_*I*_. We varied the values of the mean and standard deviation and found that the accuracy of the mean-field approximations depends on where the mean is relative to the different bifurcation regions and on the size of the standard deviation.

**Figure 3:**
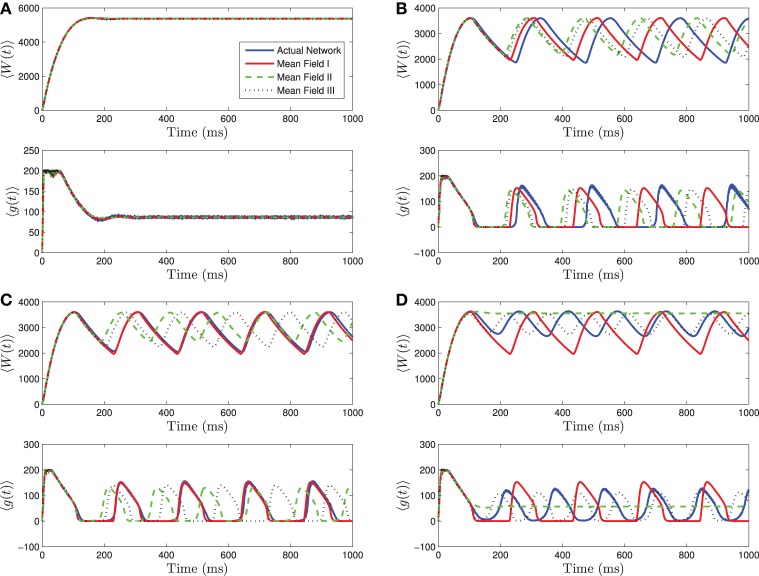
**Numerical simulations of a network of 1000 Izhikevich neurons with parameters as in Table 1, except *g*_syn_ = 200 and the applied current which is normally distributed with mean and variance as follows. (A)** 〈*I*_app_〉 = 5000 pA, σ_*I*_ = 2000 pA. **(B)** 〈*I*_app_〉 = 3000 pA, σ_*I*_ = 200 pA. **(C)** 〈*I*_app_〉 = 3000 pA, σ_*I*_ = 500 pA. **(D)** 〈*I*_app_〉 = 3000 pA, σ_*I*_ = 2000 pA. Blue is the network average of a given variable, red is MFI, green is MFII and black is MFIII. In this region, the mean-driving current is away from rheobase, 〈*I*_app_〉 » *I*_rh_. All three approximations are quantitatively and qualitatively similar for small to intermediate sized variances in the distribution of currents. For small variances, MFI is the most accurate and for larger variances, MFIII is the most accurate. For large variance, MFII bifurcates back to tonic firing earlier than MFI and MFIII, as seen in **(D)**.

As for the homogeneous network, the heterogeneous network undergoes a bifurcation from tonic firing to bursting as the amount of current applied to the individual neurons is decreased, with all other parameters held fixed. This can be seen in Figure 3 where the bifurcation with decreasing 〈*I*_app_〉 is shown. As 〈*I*_app_〉 is decreased below *I*_rh_, there is a bifurcation to quiescence. We will not discuss this latter bifurcation in detail, as we are primarily interested in analyzing the transition from tonic firing to bursting.

Note that the bifurcations described above only occur in the mean sense. Since the current values are normally distributed, there is non-zero probability that some neurons receive large enough or small enough current to be in a state other than that corresponding to the value of 〈*I*_app_〉. For small enough standard deviations, very few neurons in an actual finite network are likely to have behavior different from the mean. However, for large standard deviations, a sizable proportion may not follow the mean behavior.

Given this knowledge of the different qualitative behaviors of the network, we can see how the mean-field systems compare. For tonic firing (Figure 3A), even when the standard deviation is large, the mean-field systems approximate the network means 〈*g*(*t*)〉 and 〈*W*(*t*)〉 very well. However, when the network is bursting, with 〈*I*_app_〉 > *I*_rh_, we see a difference as to which mean-field system is superior. For small values of σ_*I*_, we have numerically found that mean-field I is superior to mean-field II and III, however all the systems are quantitatively and qualitatively accurate (see Figures 3B,C). However, for larger values of σ_*I*_, the amplitude error of MFIII is the smallest, and MFII is the worst approximation as it bifurcates to tonic firing prematurely (see Figure 3D).

**Figure 4:**
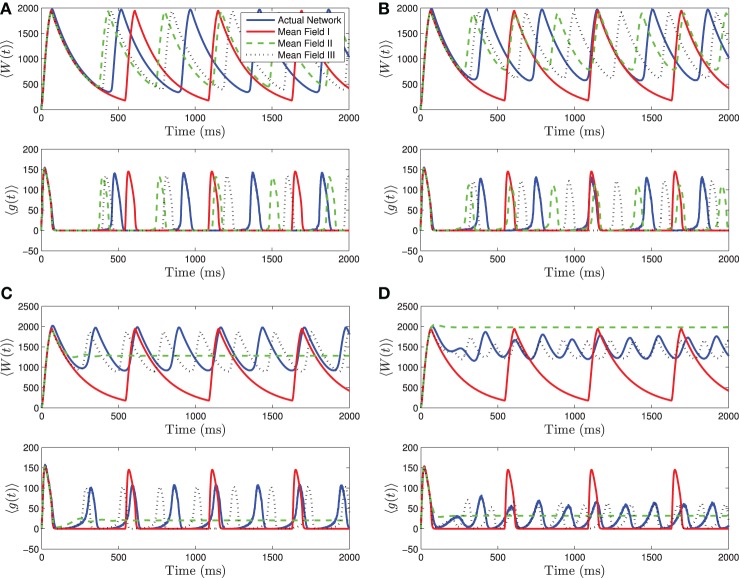
**Numerical simulations of a network of 1000 neurons with parameters as in Table 1, except *g*_syn_ = 200 and the applied current which is normally distributed with mean and variance as follows. (A)** 〈*I*_app_〉 = 1200 pA, σ_*I*_ = 200 pA. **(B)** 〈*I*_app_〉 = 1200 pA, σ_*I*_ = 500 pA. **(C)** 〈*I*_app_〉 = 1200 pA, σ_*I*_ = 1000 pA. **(D)** 〈*I*_app_〉 = 1200 pA, σ_*I*_ = 2000 pA. Blue is the network average of a given variable, red is MFI, green is MFII, and black is MFIII. In these simulations, the mean-driving current is close to (and over) the rheobase. In all cases, MFI is the least accurate. This is because it depends only on 〈*I*_app_〉. When 〈*I*_app_〉 = *O*(*I*_rh_), even for small variance, many of the neurons have *I* < *I*_rh_ and may not spike at all. **(A,B)** For small values of σ_*I*_, all three approximations are qualitatively and quantitatively accurate. **(C,D)** For larger variance, σ_*I*_ = *O*(*I*_rh_), only MFIII is qualitatively and quantitatively accurate. In this case, MFII bifurcates early to tonic firing.

When 〈*I*_app_〉 is close to *I*_rh_, we see even stronger differences between the three mean-field systems. For small to intermediate standard deviations, MFII and MFIII are clearly superior to MFI, having a smaller amplitude and frequency error (see Figures 4A,B). However, for larger values of σ_*I*_ as shown in Figures 4C,D, only MFIII is a qualitatively and quantitatively accurate representation of the behavior of the network. The amplitude and frequency error of MFI are very large, and MFII again bifurcates prematurely to tonic firing.

**Figure 5:**
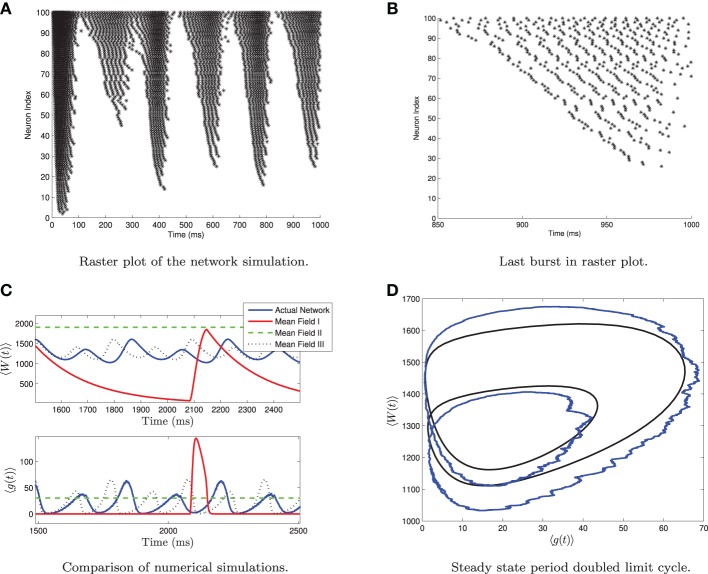
**Period doubled limit cycle in the heterogeneous network and in MFIII**. The network consists of 5000 neurons, with parameters as in Table 1, except *g*_syn_ = 200 and the applied current which is normally distributed with mean 〈*I*_app_〉 = 1100 pA and variance σ_*I*_ = 2000 pA. **(A)** Raster plot of 100 randomly selected neurons of the network arranged in order of increasing current. Individual neuron behaviors include burst firing, alternate burst firing, tonic firing and quiescence. **(B)** Close-up of raster plot. The neurons appear to fire in “traveling waves” during a burst. **(C)** Comparison of the mean variables of the large network simulation and the simulations of the mean field systems. Only MFIII is able to reproduce the period doubling behavior. **(D)** Comparison of the “phase portrait” of period doubled limit cycle for MFIII and the mean variables of network.

One should note that for 〈*I*_app_〉 = *O*(*I*_rh_) and for large values of σ_*I*_, the network can undergo a period doubling bifurcation. This is shown in Figure 5. The large standard deviation in the current forces different neurons into different regimes, such as tonic firing, bursting, alternate burst firing and quiescence as seen in Figure 5A. During a burst the heterogeneity causes the neurons to fire asynchronously. Neurons with higher applied current fire followed by those with lower applied current. See Figure 5B. A small subpopulation of neurons with low applied current are alternate bursters (i.e., burst with twice the period of the rest of the bursting neurons). This appears as a period doubled limit cycle in the mean variables of the network, as seen in Figures 5C,D. Only MFIII is able to approximate the period-doubled limit cycle with any degree of accuracy, as shown in Figures 5C,D. Period doubling bifurcations are well known for their capability of inducing chaos. Given that MFIII accurately represents the period doubling bifurcation, it may be able to replicate any potential chaotic behavior. However, we leave further investigation of this interesting behavior for future work.

To summarize, all the mean-field systems are valid for tonic firing parameter regimes, and MFI is valid for all parameter regimes with small σ_*I*_, except for 〈*I*_app_〉 = *O*(*I*_rh_). Mean-Field II and III are valid for bursting with 〈*I*_app_〉 » *I*_rh_, and MFIII is the only valid approximation for 〈*I*_app_〉 = *O*(*I*_rh_). Thus, when 〈*I*_app_〉 is large we may be able to use MFII to determine the type of bifurcation(s) involved when a heterogeneous network transitions from tonic firing to bursting and the location in parameter space of the bifurcation curves. Note that when the mean network behavior undergoes a bifurcation from a tonic firing steady state to a bursting oscillation, this does not indicate that the entire network of neurons is bursting, or tonically firing. However, we will show how to use MFIII to determine what proportion of neurons display the different types of behavior, given a specific parameter regime and level of heterogeneity.

In addition to simple heterogeneity using unimodal distributions, one can also apply the same three mean-field equations to networks where multiple subpopulations exist. However, unlike previous attempts at modeling networks with multiple subpopulations, we do not generate discrete coupled subnetworks with different fixed values of the parameters in each subnetwork. Instead we use a smoother approach where the networks have distributions of parameters with multiples modes indicative of multiple subpopulations. This can be easily done through the processing of mixing unimodal distributions (see Appendix C).

### 3.2. Applications of mean-field theory with a single source of heterogeneity

#### 3.2.1. Numerical bifurcation analysis using MFII

As shown in Figure 3 the CA3 model network a makes transition from tonic firing to bursting as 〈*I*_app_〉 is varied. Similar transitions occur when *g*_syn_ is varied. In this section, we use numerical bifurcation analysis of MFII to determine the bifurcations involved in this transition, and the curves where they occur in the 〈*I*_app_〉-*g*_syn_ parameter space. Since the mean-field system (Equations 50–54) consists of switching ODEs, this involves bifurcations of non-smooth systems as well as standard (smooth) bifurcations. A review of the theory of non-smooth systems can be found in di Bernardo et al. (2008). For the standard (smooth) bifurcations, the numerical bifurcation analysis is done in MATLAB (MATLAB, 2012) using the MATCONT package (Dhooge et al., 2003). While it is possible to apply typical numerical continuation techniques to non-smooth bifurcations, primarily by defining alternate sets of test functions, and functions defining non-smooth limit cycles and equilibria for continuation (see Kuznetsov et al., 2003), implementation of these algorithms is outside of the scope of this paper. We opted instead to determine the non-smooth bifurcations via direct numerical simulations. We compare the mean field theory results to those for the homogeneous system and to direct simulations of large networks.

**Figure 6:**
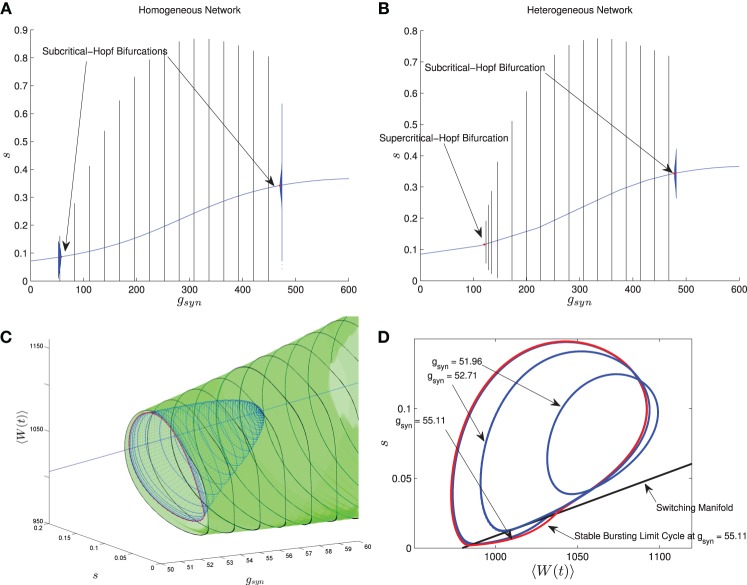
**Comparison between the bifurcation structure of homogeneous and heterogeneous networks using mean-field models**. The parameters are as in Table 1, except the applied current which is normally distributed with mean and variance as follows and *g*_syn_ which varies as shown. **(A)** MFI, *I*_app_ = 2000 pA. **(B)** MFII, 〈*I*_app_〉 = 2000 pA, σ_*I*_ = 500 pA. **(C)** MFI, low *g*_syn_ bifurcation sequence (3D view). **(D)** MFI, low *g*_syn_ bifurcation sequence, (2D view). In **(A,B)**, the curved blue lines denote the value of the equilibrium point, which corresponds to tonic firing in the network. The vertical black/blue lines denote the amplitude range for the stable/unstable limit cycles, respectively. These correspond to bursting if the amplitude reaches zero, otherwise they correspond to the network having an oscillatory average firing rate. **(A)** Homogeneous case. Numerical bifurcation analysis of MFI displays two subcritical Hopf bifurcations: one at a low *g*_syn_ value and one at a high value. **(B)** Heterogeneous case. Numerical bifurcation analysis of MFII also displays two Hopf bifurcations, but the one at the low *g*_syn_ value is supercritical. This makes bursting at low *g*_syn_ values less robust in the heterogeneous case as discussed in the text. The non-smooth sequence of bifurcations for the homogeneous network is expanded upon in **(C,D)**. In **(C)**, we continue the smooth unstable limit cycle (blue) from the Hopf bifurcation at low *g*_syn_ values until the continuation halts (red). This occurs close to the grazing bifurcation with the switching manifold. We use direct numerical simulations to continue the stable non-smooth limit cycle (green). Key points in this bifurcation sequence are shown in the phase plane in **(D)**. A smooth unstable limit cycle expands and grazes the switching manifold at approximately *g*_syn_ = 52.71 nS, and persists to collide in a non-smooth saddle-node of limit cycles at approximately *g*_syn_ = 55.11 nS. This sequence of bifurcations happens very rapidly in the parameter space, leaving a narrow region of bistability between the tonic firing and bursting solutions. Note that the totally unstable-non smooth limit cycles in **(D)** are computed via direct simulation of the time reversed MFII system.

In Nicola and Campbell (2013) we carried out a numerical bifurcation analysis for a homogeneous network. The mean-field equations in this case, which are the same as MFI with 〈*I*_app_〉 replaced by *I*_app_, indicate that the transition from tonic firing to bursting occurs via the following sequence of bifurcations. The stable bursting limit cycle is created in a saddle node bifurcation of (non-smooth) limit cycles. The smaller, unstable limit cycle becomes smooth in a grazing bifurcation and then disappears in a subcritical-Hopf bifurcation which destabilizes the equilibrium point corresponding to tonic firing. This transition is shown in Figure 6A when *I* is held fixed and *g*_syn_ is varied. The bursting limit cycles are created at a low *g*_syn_ value and destroyed at a high *g*_syn_ value. Details of the sequence of bifurcations for low *g*_syn_ are illustrated in Figures 6C,D. The sequence for high *g*_syn_ is the same but reversed.

**Figure 7:**
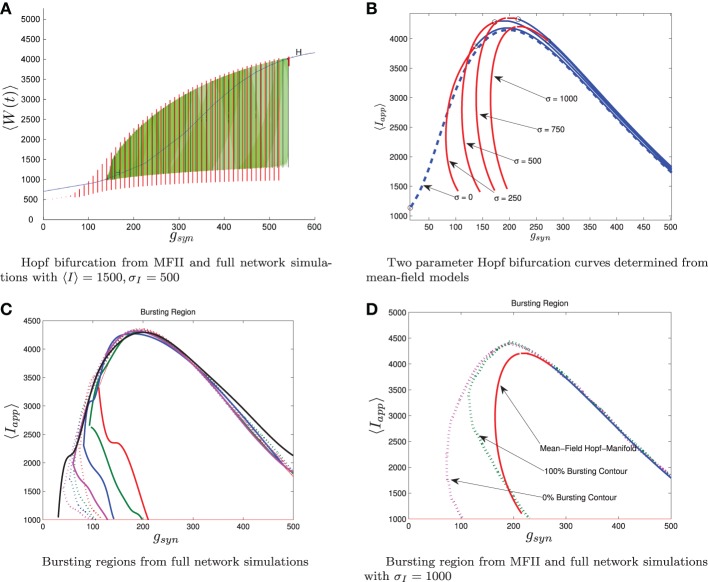
**Comparison between numerical bifurcation analysis of MFII and direct simulation of the full network**. The parameters are as in Table 1, except *g*_syn_ varies as discussed below and the applied current which is normally distributed with mean and variance as discussed below. **(A)** Simulations of a network of 10,000 neurons with 〈*I*_app_〉 and σ_*I*_ as shown were run at discrete values of *g*_syn_ for 2000 ms. The last 400 (ms) of simulation time is plotted (in red), showing the stable limit cycle oscillation for different *g*_syn_ values. This is compared to numerical continuation of the MFII limit cycle and equilibrium (in green and blue). Both the actual network and MFII appear to undergo a supercritical Hopf bifurcation for low *g* values and a subcritical Hopf for high *g* values. **(B)** The Hopf bifurcation curves for the mean-field systems with σ_*I*_ as shown. Red denotes supercritical Hopf bifurcations and blue denote subcritical Hopf bifurcations. The black circles denote codimension 2 Bautin bifurcation points. **(C)** Simulations of a network of 1000 neurons run on a discrete mesh of 〈*I*_app_〉 and *g*_syn_ values. The 0% (dotted line) and 100% (solid line) network bursting contours for σ_*I*_ = 0, 250, 500, 750, and 1000 pA are colored in black, magenta, blue, green, and red, respectively. The curves are spline fits to the actual contours. **(D)** MFII Hopf bifurcation curves and spline fits to the 0% bursting and 100% bursting contours of the actual network for σ_*I*_ = 1000.

Using MFII, we numerically confirm that, as for the homogeneous network, the mean-field system of the heterogeneous network undergoes a Hopf bifurcation as the network transitions from tonic firing to bursting. However, as shown in Figure 6B, with 〈*I*_app_〉 held fixed the transitions for low *g*_syn_ and high *g*_syn_ are not the same. For high *g*_syn_ the transition is the same as the homogeneous case. For low *g*_syn_ the transition occurs via the following sequence of bifurcations. A supercritical Hopf bifurcation destabilizes the equilibrium point corresponding to tonic firing and creates a stable limit cycle. This limit cycle is smooth and hence corresponds not to bursting, but to firing with an oscillatory firing rate. This limit cycle then grows until it becomes a non-smooth, bursting limit cycle in a grazing bifurcation (in Figure 6B this occurs at *g*_syn_ ≈ 150). We verified this prediction of the mean-field model by running direct simulations of a network of 10,000 neurons with fixed 〈*I*_app_〉, σ_*I*_ while varying the *g*_syn_ value. As shown in Figure 7A, when the steady state mean variables are plotted vs *g*_syn_, the supercritical nature of the Hopf bifurcation in the large network is apparent.

To further investigate the heterogeneous case, we used MATCONT to carry out two parameter continuation of the Hopf bifurcation for MFII with four different values for the standard deviation of *I*_app_: σ_*I*_ = 250,500,750, and 1000 pA. As shown in Figure 7B, in all cases there appears to be a codimension-2 Bautin (or generalized Hopf) bifurcation on the two-parameter Hopf bifurcation curve, with the Hopf being supercritical on the left boundary before this point and subcritical after. By contrast, in the homogeneous case (σ_*I*_ = 0 line in Figure 7B) the bifurcation is subcritical everywhere on the two-parameter Hopf curve.

Further verification of the mean-field results can be found in the direct numerical simulations of a network of 500 neurons shown in Figure 7C. The simulations were run on a 50 × 50 mesh in the *g*_syn_ vs 〈*I*_app_〉 parameter space, using five different values for the standard deviation of *I*_app_: σ_*I*_ = 0, 250,500,750, and 1000 pA. Note that σ_*I*_ = 0 is the homogeneous network. The proportion of bursting neurons, *p*_burst_, was computed using Equation (A6) (see Appendix B). The 0 and 100% bursting contours can be seen in Figures 7C,D. In these figures, there appear to be two kinds of transitions from tonic firing to bursting. Along the (lower) left part of the boundary of the bursting region, the transition is gradual: the proportion of bursting neurons gradually increases from 0 to 100%. Along the rest of the boundary, however, the whole network transitions to bursting simultaneously. This agrees with the prediction from the mean-field model that two different bifurcations occur along the bursting boundary. Note also that the size of the entire bursting region and the 100% network bursting region get smaller as the level of heterogeneity (σ_*I*_) increases.

Let us reiterate the primary differences between supercritical and subcritical Hopf induced bursting seen in Figures 6, 7. First, the subcritical case allows for bursting a lower *g*_syn_ values than the supercritical case. This is because in the subcritical case bursting is initiated via a a saddle-node of limit cycles bifurcation which occurs to the *left* of the Hopf bifurcation, while in the supercritical case, bursting starts to the *right* of the Hopf in a grazing bifurcation. Second, the transition to bursting is sharp in the subcritical case and gradual in the supercritical case. The supercritical Hopf bifurcation is consistent with the gradual transition from bursting to firing seen in Figures 7C,D. When only a few neurons are bursting and the rest have oscillatory firing rates the corresponding mean behavior is a limit cycle with small amplitude. As more and more neurons become bursting this increases the amplitude of the limit cycle of the mean behavior until it grazes the switching manifold. In the subcritical case, the saddle-node of limit cycles involves large amplitude limit cycles, corresponding to all the neurons being in the bursting state.

The bifurcation curves in Figure 7B are both qualitatively and quantitatively accurate descriptions of the behavior of the actual network. For example, in the actual network simulation, the bursting region decreases as σ_*I*_ increases (see Figure 7C). This same behavior is displayed by the MFII equations, albeit to a greater degree, as shown in Figure 7B. However, there is a greater degree of quantitative error for lower values of *g*_syn_ and larger values of σ_*I*_. In particular, for a fixed value of σ_*I*_ MFII predicts that the Hopf bifurcation occurs at a higher value of *g*_syn_ than occurs in the real network (compare Figures 7B,D directly) and this prediction error seems to increase as σ_*I*_ increases. This is why MFII indicates the network should be tonically firing when σ_*I*_ is high (in Figure 3D, for example).

Taken together, these results indicate that for small *g*_syn_, network induced bursting via adaptation is not robust to heterogeneity in the applied current. This occurs for qualitative and quantitative reasons, both related to the Hopf bifurcation associated with the left boundary of the bursting region. Qualitatively, the addition of heterogeneity causes this bifurcation to change from subcritical to supercritical making the bursting less robust for small *g*_syn_ values. Quantitatively, the *g*_syn_ value of this bifurcation increases when the heterogeneity becomes stronger, while the value of the Hopf bifurcation associated with the right boundary does not change appreciably. Thus the size of the bursting region decreases with increasing heterogeneity.

#### 3.2.2. Bifurcation types and manifolds using MFIII

It is difficult to use MATCONT with MFIII as MFIII is an infinite dimensional dynamical system, as it is a PDE. However, the existence of equilibrium points can be determined using standard root finding algorithms. While direct bifurcation analysis is difficult to implement in this situation, one can use properties of the firing rate to describe, qualitatively and quantitatively, any transitions between network states. This will be the approach of this section. To begin, we consider networks that are tonically firing, we then proceed to the study of bursting networks.

For a network of neurons with heterogeneity in the parameters, even if all the neurons are tonically firing, one cannot find a steady state firing rate for the network, as in the case of a homogeneous network. The parameter heterogeneity creates a distribution of steady state firing rates across the network. While the mean-field equations by themselves can only determine the mean of this distribution, with an added assumption we can approximate the distribution of steady state firing rates for the network with a great degree of accuracy.

Consider a network with just one heterogeneous parameter, β. Assume that the steady state firing rate of each neuron in the network can be related to its value for the heterogeneous parameter: *R*_*i*_ = *g*(β). Assume further that one can approximate this function by the steady state value of 〈*R*_*i*_(*t*)|β〉:

(60) $$g(\beta )\approx \langle R_{i}|\beta \rangle .$$

This is easily determined through direct simulation of MFIII, Equations (56–58), until the system reaches steady state. Treating *g* as the transformation of a random variable, one can determine the steady state distribution of firing rates in the network, ρ_*R*_(*r*), through the standard theorem on transforming random variables:

(61) $$\rho_{R}(r)=\rho_{\beta }(g^{-1}(r))\left|\frac{d}{dr}g^{-1}(r)\right|.$$

which can be found in any standard textbook on probability theory (such as Renyi, 1970). Note that we must assume that 〈*R*_*i*_|β〉 is monotonic and invertible for this procedure to be valid.

**Figure 8:**
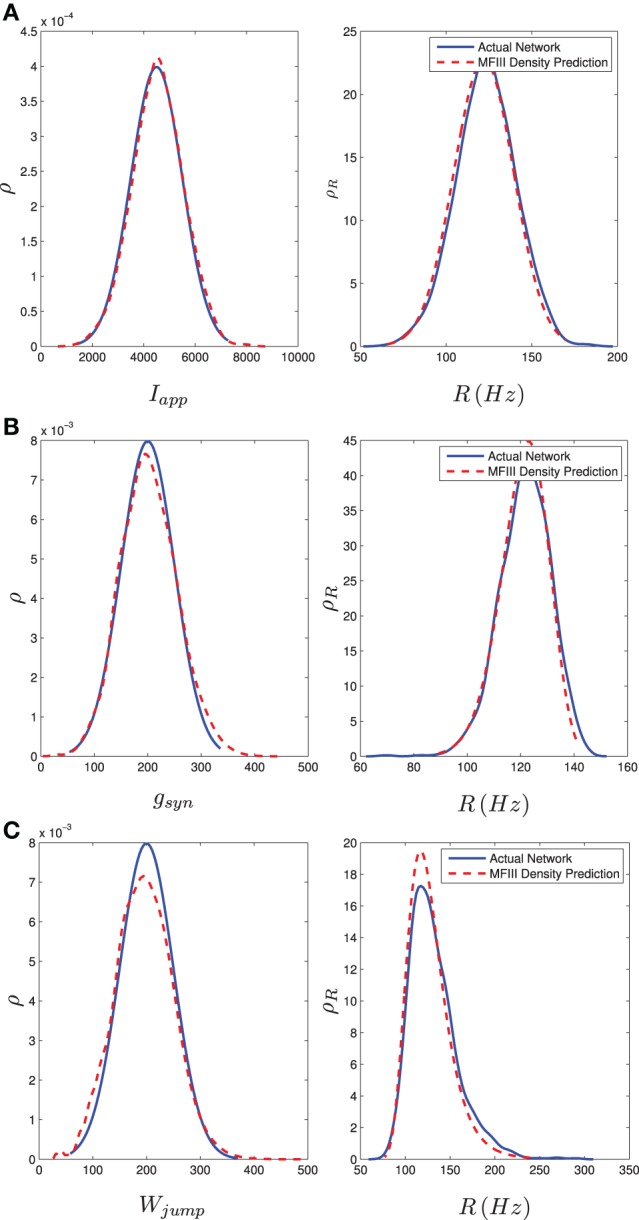
**Heterogeneity in *I*_app_, *g*_syn_, or *W*_jump_ leads to heterogeneity in the firing rate**. As described in section 3.2.2, given the parameter distribution (solid curves, left column) MFIII can be used to estimate the corresponding distribution of firing rates in the network (dashed curves, right column). As described in section 3.2.3, given the steady state firing rate distribution of an actual network (solid curves, right column) MFIII can be used to estimate the parameter distribution in the network (dashed curves, left column). The network firing rate distribution is estimated using a histogram. The calculations were carried out on a network of 1000 neurons. Parameters, other than those given below, can be found in Table 1. **(A)** Distribution of *I*_app_ with 〈*I*_app_〉 = 4500 pA, σ_*I*_ = 1000 pA. **(B)** Distribution of *g*_syn_ with 〈*g*_syn_〉 = 200 nS, σ_*g*_ = 50 nS. **(C)** Distribution of *W*_jump_ with 〈*W*_jump_〉 = 200 pA, σ_*W*_ = 50 nS.

We carried out this computation for a network of 1000 neurons with a normal distribution in either *I, g*, or *w*_jump_. Details of the implementation can be found in Appendix D. We numerically determined the distribution of steady state firing rates for the neurons in the full network through

(62) $$R_{i}=\frac{1}{ISI_{i,\text{last}}},\;i=1,2,\ldots N$$

where *ISI*_*i*, last_ is the last interspike interval for the *i*th neuron measured from a lengthy (1000 ms) simulation. Figure 8 shows the results of the two approaches. The blue curve in the left column shows the distribution of parameter values. This is used in MFIII to calculate the predicted distribution of firing rates, which is the dashed red curve in the right column. The solid blue curve in the right column is the computed distribution of firing rates from numerical simulation of the full network equations. There is excellent agreement between the firing rate distributions in all cases.

**Figure 9:**
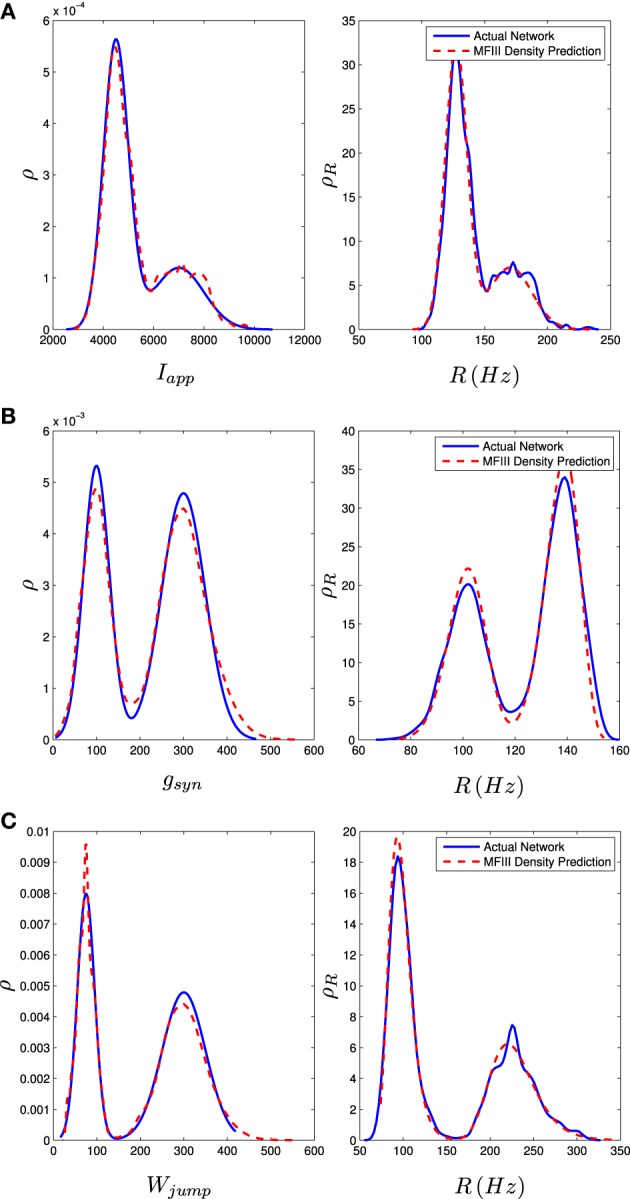
**Bimodal distributions in *I*_app_, *g*_syn_, and *W*_jump_ lead to bimodal distributions in the firing rate**. These bimodal parameter distributions are generated through distribution mixing of two normal subpopulations with standard deviations and means as given below. See Appendix C for details. The distribution of the firing rate or the distribution of the parameter can be computed using MFIII if one knows the complementary distribution. See sections 3.2.2 and 3.2.3 for details. Curve descriptions are as given in Figure 8. Parameters, other than those given below, can be found in Table 1. The calculations were carried out on a network of 1000 neurons. **(A)** Distribution of *I*_app_ with μ_1_ = 4500 pA, σ_1_ = 500 pA, μ_2_ = 7000 pA, σ_2_ = 1000 pA, *m* = 0.7. **(B)** Distribution of *g*_syn_ with μ_1_ = 100 nS, σ_1_ = 30 nS, μ_2_ = 300 nS, σ_2_ = 50 nS, *m* = 0.4. **(C)** Distribution of *W*_jump_ with μ_1_ = 300 pA, σ_1_ = 50 pA, μ_2_ = 75 pA, σ_2_ = 20 pA, *m* = 0.6.

We carried out the same computations with a bi-modal distribution in *I, g*, or *w*_jump_, generated by mixing normal unimodal distributions (see Appendix C). This is one way of representing a network with two subpopulations of neurons with different parameters. The mean field approach again gives an excellent approximation to the qualitative and quantitative properties of the steady state distribution of firing rates, as shown in the right column of Figure 9.

The above approach is only valid when the network is tonically firing. In this situation the steady firing rates of the network and the individual neurons are constant. When the network leaves the tonic firing regime, however, these steady state firing rates become oscillatory. In the case of bursting, the amplitude of the oscillation is large enough that the firing rate goes to zero for intervals of time. Whether or not the neurons are bursting, oscillatory firing rates cannot be represented as a simple distribution of firing rates. However, with some additional work we can use the tools developed above to determine what proportion of neurons in the network is bursting. This is a statistical alternative to direct bifurcation analysis.

**Figure 10:**
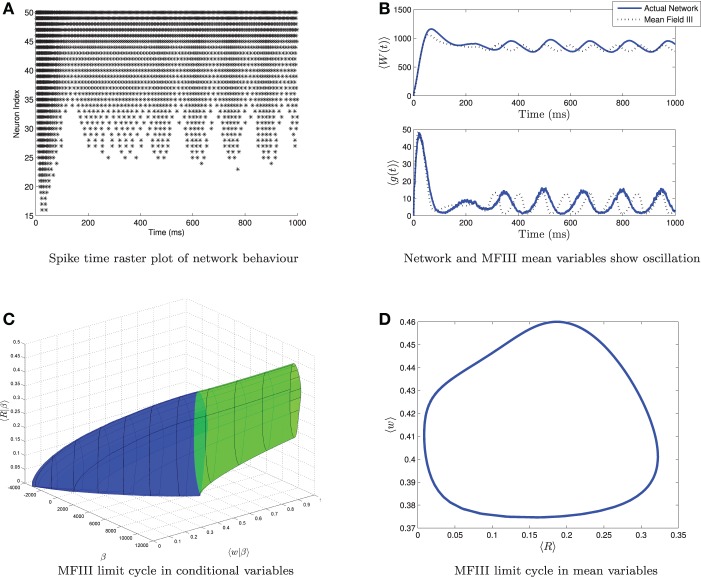
**Visualizing a limit cycle in a heterogeneous network**. Numerical simulation of MFIII and a network of 1000 neurons with heterogeneity in the applied current. Parameters are as given in Table 1 except *g*_syn_ = 200 nS, 〈*I*_app_〉 = 1000 pA and σ_*I*_ = 4400 pA. **(A)** Raster plot for 50 randomly selected neurons of the network arranged in order of increasing current. Some of the neurons are bursting, while others are tonically firing, albeit with an oscillatory firing rate. **(B)** In the mean variables, the steady state behavior of both the network and MFIII is an oscillation. **(C)** As MFIII is a partial differential equation, the steady state “limit cycle” is actually a manifold of limit cycles, foliated by the heterogeneous parameter β = *I*_app_. Part of the manifold has cycles with 〈*R*|β〉 = 0 for an extended period of time (in blue). The other part contains limit cycles that have 〈*R*|β〉 ≠ 0 during the entire oscillation. We can classify neurons with the parameter values in blue as bursting, and those in green as oscillatory firing. **(D)** Averaging the limit cycle in **(C)** with respect to β yields the mean limit cycle.

From simulations of the full network, we know that when the variance in the heterogeneity is large enough, not all of the neurons necessarily display the behavior predicted from the mean-field equations. For example, Figure 10 shows simulations of a network where the mean-field equations display an oscillatory firing rate which does not quite go to zero. The spike time raster plot of the full network (Figure 10A) shows that some neurons *are* bursting while others are tonically firing with an oscillatory firing rate. However, simulation of the corresponding mean-field equations (Figure 10B, dashed line) reveals only an oscillation, not bursting. While this is consistent with the behavior of the network mean variables (Figure 10B, solid line), we have lost the information that some of the neurons are bursting. Similarly, one can find examples where the mean-field equations exhibit bursting, but not all neurons in the network are bursting. Thus, it would be useful to have more information about individual neuron behavior. MFIII can be used to obtain such information.

In the situation described above, the steady state mean network firing rate is oscillatory. We will denote this oscillatory solution as γ. We interpret γ to be the limit cycle parameterized by γ(*t*) with (*s*(γ(*t*)), 〈*w*(γ(*t*))|β〉) being the graph of the limit cycle in phase space. We will denote the period of the limit cycle as *T*. In this case, the “steady state” firing rate for neuron *i* will be a periodic function of time: *R*_*i*_(*t*), which depends on γ and the value of the parameter β associated with the neuron: *R*_*i*_(*t*) = *g*(β, γ(*t*)), for *t* ∈ [0, *T*]. To proceed, we make the same assumption as above, that

(63) $$g(\beta ,\gamma (t))\approx \langle R_{i}(\gamma (t))|\beta \rangle$$

where 〈*R*_*i*_(γ(*t*))|β〉 is the oscillatory firing rate associated with the steady state limit cycle γ in MFIII. An example of the graph of the steady state limit cycle derived from MFIII is shown in Figure 10C. In this visualization we can clearly see that part of the network is bursting (blue) while the rest is tonically firing with an oscillatory firing rate (green). Integration of this limit cycle over the heterogeneous parameter returns the “mean” limit cycle (Figure 10D).

We now use this setup to approximate *p*_burst_, the proportion of neurons in the network that are bursting during the network level oscillation γ. Noting that 〈*R*_*i*_(γ(*t*)|β)〉 ≥ 0, for all *t* ∈ [0, *T*], the tonically firing neurons correspond to those β values for which 〈*R*_*i*_(γ(*t*))|β〉 > 0, for all *t* ∈ [0, *T*]. Thus we define, *p*_tonic_, the proportion of tonically firing neurons in the network via

(64) $$p_{\text{tonic}}=\int_{\beta }X\left(\left[\underset{t\in [0,T]}{\min}\langle R_{i}(\gamma (t))|\beta \rangle \right]>0\right)\rho_{\beta }(\beta )d\beta$$

where *X* is the usual indicator function. Similarly, the proportion of quiescent (non-firing) neurons is given by

(65) $$p_{q}=\int_{\beta }X\left(\left[\underset{t\in [0,T]}{\max}\langle R_{i}(\gamma (t))|\beta \rangle \right]=0\right)\rho_{\beta }(\beta )d\beta .$$

Recall that the bursting neurons correspond to those β values such that 〈*R*(γ(*t*))|β〉 = 0, for some subinterval of [0, *T*]. Thus we must have

(66) $$p_{\text{burst}}=1-p_{q}-p_{\text{tonic}}.$$

We compute these values as follows. First we numerically integrated MFIII until the steady state oscillation γ is reached. This is an oscillation of 〈*w*(*t*)|β〉 and *s*(*t*). The corresponding oscillatory firing rate 〈*R*_*i*_ (γ(*t*))|β〉 is computed through Equation (58) as a function of β on the limit cycle γ. One can then determine

(67) $$m(\beta )=\underset{t\in [0,T]}{\min}\langle R(\gamma (t))|\beta \rangle ,$$

(68) $$M(\beta )=\underset{t\in [0,T]}{\max}\langle R(\gamma (t))|\beta \rangle ,$$

and the integrals simplify to

(69) $$p_{\text{tonic}}=\int_{\beta }h(m(\beta ))\rho_{\beta }(\beta )d\beta$$

(70) $$p_{q}=\int_{\beta }h(-M(\beta ))\rho_{\beta }(\beta )d\beta$$

where *h* is the Heaviside function, with *h*(0) = 1.

**Figure 11:**
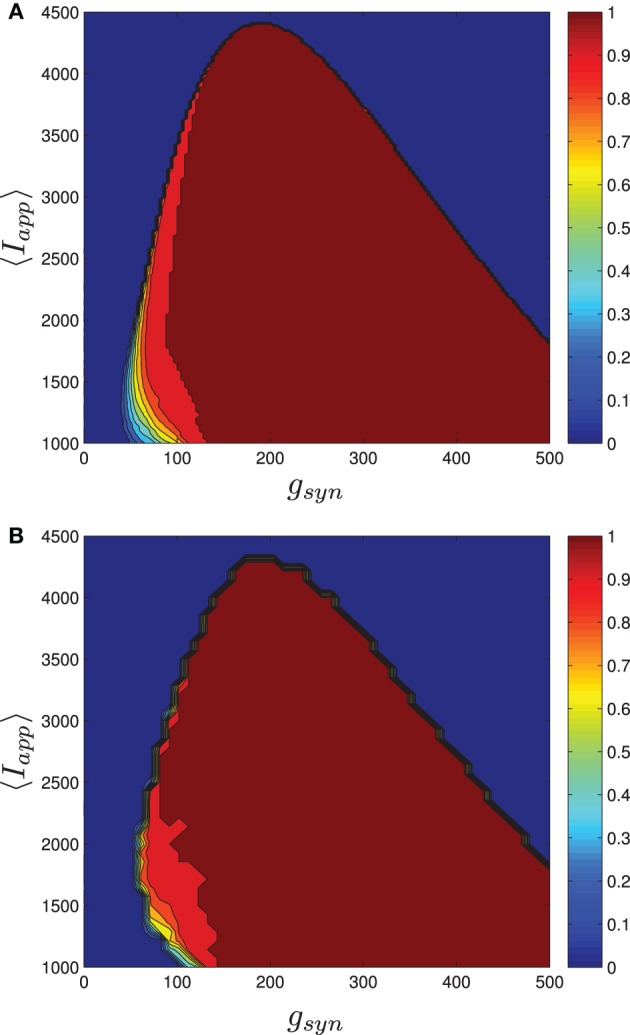
**The proportion of bursting neurons, *p*_burst_ for MFIII and an actual network. (A)** Using the techniques outlined in the text, MFIII is used to compute the proportion of bursting neurons, *p*_burst_ at each point in a mesh over the parameter space. **(B)** Numerical simulations of a network of 500 neurons are used to compute the proportion of bursting neurons, *p*_burst_. All the parameters for both the MFIII system and the actual network are identical (see Table 1), except that MFIII is run over a finer mesh. The results using MFIII are both qualitatively and quantitatively accurate. **(A)** MFIII, σ_*I*_ = 500 pA. **(B)** Network, σ_*I*_ = 500 pA.

Numerical results for a network with single heterogeneous parameter are shown in Figure 11. For Figure 11A, the proportion of bursting neurons was computed, using the method described above, at each point in a mesh on the *g*_syn_ vs. 〈*I*_app_〉 parameter space. This data was then used to generate the *p*_burst_ contours. For Figure 11B the actual network was simulated to steady state at each point of a slightly coarser mesh. The proportion of bursting neurons at each point was computed according to Equation (A6) in Appendix B and used to generate the *p*_burst_ contours. The results of the mean-field computation are both qualitatively and quantitatively accurate. In particular, MFIII recovers the gradual transition to bursting on the left boundary of the bursting region and the abrupt transition to bursting on the right. It should be noted that it is much faster, by approximately an order of magnitude, to run a mesh of integrations over MFIII then it is to run mesh over an actual network.

#### 3.2.3. Inverting a steady state firing distribution to determine the distribution of parameters using MFIII

Many parameters for neuron models are difficult to measure directly using electrophysiology. However, a distribution of firing rates across a network of neurons is relatively easy to measure using intracellular recordings, or can be estimated using measurements from multi-electrode recordings and spike sorting algorithms, among other methods (Buzsáki, 2004; Grewe et al., 2010). We have seen in the previous section that, given a distribution of heterogeneities, MFIII can predict the steady state distribution of firing rates. Here we show that one can invert this process to yield a distribution of parameters given a steady state distribution of firing rates.

We assume that only the firing rate distribution is known, and denote it ρ_*R*_(*r*) as above. We then proceed as in the previous section, assuming that the steady state firing rate for a particular neuron is some function of the heterogeneous parameters *R*_*i*_ = *g*(β) and that this function is well approximated by 〈*R*_*i*_|β〉. Under these assumptions, one can solve for the distribution of parameters β using

(71) $$\rho_{\beta }(\beta )=\rho_{R}(g(\beta ))\left|\frac{d}{d\beta }g(\beta )\right|$$

which follows from standard statistical theorems on the transformations of random variables (Renyi, 1970). Note that we need to assume that 〈*R*_*i*_|β〉 is differentiable for this procedure to be valid.

The primary problem we face in using this approach to approximate the distribution ρ_β_(β) is that we need to determine the steady state values of the function 〈*R*_*i*_|β〉. However, a cursory look at the equations for MFIII shows that these in fact depend on ρ_β_(β), the function we are trying to find, through the equation for *s*:

(72) $$\dot{s}=-\frac{s}{\tau_{s}}+s_{\text{jump}}\int_{\beta }\langle R_{i}(t)|\beta \rangle \rho_{\beta }(\beta )d\beta .$$

Fortunately, however, this problem disappears when we look at the steady state value for *s*:

(73) $$\bar{s}=\tau_{s}s_{\text{jump}}\int_{\beta }\langle R_{i}|\beta \rangle \rho_{\beta }(\beta )d\beta =\tau_{s}s_{\text{jump}}\langle R_{i}\rangle .$$

Here 〈*R*_*i*_〉 is the unconditioned steady state mean of the firing rate distribution. This information is readily available, as we have assumed we know the steady state distribution, ρ_*R*_(*r*), and determining the first moment is numerically trivial.

Putting the expression for *s* into the steady state equation for 〈*w*|β〉 yields a set of coupled equations:

(74) $$\langle w|\beta \rangle =\tau_{w}w_{\text{jump}}\langle R_{i}|\beta \rangle ,$$

(75) $$\langle R_{i}|\beta \rangle =\{\begin{array}{ll} {\left[\int_{V}\frac{dv}{G_{1}(v,\tau_{s}s_{\text{jump}}\langle R\rangle ,\langle w|\beta \rangle ,\beta )}\right]}^{-1} & :H(\langle w|\beta \rangle ,\bar{s},\beta )\geq 0\\ 0 & \;H(\langle w|\beta \rangle ,\bar{s},\beta )<0 \end{array}$$

These may be solved for 〈*w*|β〉 and 〈*R*_*i*_|β〉 by discretizing in β and numerically solving the resulting system at each grid point with any standard root finding algorithm.

Alternatively, one can set *s* to its equilibrium value in MFIII and numerically integrate the resulting equation:

(76) $${\langle w|\beta \rangle }^{\prime }\;=-a\langle w|\beta \rangle +w_{\text{jump}}\langle R_{i}(t)|\beta \rangle ,$$

(77) $$\langle R_{i}(t)|\beta \rangle =\{\begin{array}{ll} {\left[\int_{V}\frac{dv}{G_{1}(v,\tau_{s}s_{\text{jump}}\langle R_{i}\rangle ,\langle w|\beta \rangle ,\beta )}\right]}^{-1} & :H(\langle w|\beta \rangle ,\bar{s},\beta )\geq 0\\ 0 & \;H(\langle w|\beta \rangle ,\bar{s},\beta )<0 \end{array}$$

until it reaches steady state, which will determine 〈*w*|β〉 and 〈*R*_*i*_|β〉. Note that this approach will only work if the tonic firing equilibrium of the original mean-field system MFIII is asymptotically stable.

We have implemented this approach as follows. A network of 1000 neurons is numerically integrated until it reaches its steady state firing rate. The distribution of firing rates over the network is found as described in the previous section. The density function for this distribution, ρ_*R*_(*r*), is then estimated using the firing rate histogram. Equations (76), (77) are numerically integrated until they reach steady state. We then substitute the estimate of ρ_*R*_(*r*) and the approximation 〈*R*_*i*_|β〉 of *g*(β) into (71) to determine the parameter distribution ρ_β_(β). See Appendix D for more details. Our results for unimodal and bimodal distributions are shown in Figures 8, 9, respectively. In the right column of each figure, the solid blue curve is the distribution of steady state firing rates from integration of the full network. In the left column of each figure the dashed red curve is the estimate of ρ_β_(β) found using the procedure above, while the blue curve is the actual parameter distribution used in the network simulation. We note that no information about the distribution of parameters is known in the estimation procedure, yet the numerical results are very accurate in both the unimodal (Figure 8) and the bi-modal case (Figure 9).

Perhaps most interesting is that we can extend this technique to estimate the individual neuron parameter values, β_*i*_, *i* = 1, …, *N*. This again follows from the assumption that *g*(β) = 〈*R*_*i*_|β〉 is the function that transforms the random variables β_*i*_ into *R*_*i*_. If the function is invertible, then we can compute the individual β_*i*_ through numerically inverting the steady state 〈*R*_*i*_|β〉. For example, when this technique is applied to a network where the only source of heterogeneity is *I*, the mean relative absolute error in the predicted values *Î*_*i*_ versus the actual values *I*_*i*_ is only 0.6%. The details about how to numerically invert for the individual parameter values are included in Appendix D.

While network level inversion of a single heterogeneous parameter is an important step forward, this is performed under very strong assumptions. In particular, when performing this inversion, all of the heterogeneity in the firing rates is assumed to come from a single parameter. Additionally, all the other parameters are assumed to be known. These two assumptions are exceptionally strong and one has to take great care in inverting actual recorded firing rates from neurons that they be reasonably satisfied.

### 3.3. Mean-field applications with multiple sources of heterogeneity

In order for the mean-field applications to be useful for realistic neuronal networks, one needs to consider heterogeneity in more than one parameter. Recall that the mean field systems derived in section 2.3 are valid for multiple heterogeneous parameters, one simply considers β to be a vector instead of scalar. This presents some difficulties in implementation which we discuss in the section. The examples we consider will have 2 or 3 sources of heterogeneity, primarily in the parameters *I, g* and *d*.

Recall that MFII is given by the Equations (50–54). The main difficulty in dealing with MFII lies with the integral terms, which are now multiple integrals. For example:

(78) $$\langle R_{i}\rangle =\int_{\beta_{1}}\int_{\beta_{2}}\ldots \int_{\beta_{p}}\langle R_{i}|\beta \rangle \rho_{\beta }(\beta_{1},\beta_{2},\ldots \beta_{p})d\beta_{p}\ldots d\beta_{2}\beta_{1}$$

where *p* is the number of heterogeneous parameters. In order to numerically integrate or carry out bifurcation analysis on MFII, these multiple integrals must be evaluated. We have found that this is most easily done using a Monte–Carlo numerical integration scheme. Once this is implemented, bifurcation diagrams can be generated exactly as for the case of one parameter heterogeneity: the equilibrium points and smooth limit cycles are continued using MATCONT, while the non-smooth limit cycles are generated using numerical simulations.

The integral term in MFIII can be dealt with in a similar way as to that for MFII. Once this is implemented, the steady states and network properties can be determined as described in section 3.2, while numerical simulations can be used to follow stable periodic solutions. We will use this approach later on in our case study on adaptation induced bursting.

**Figure 12:**
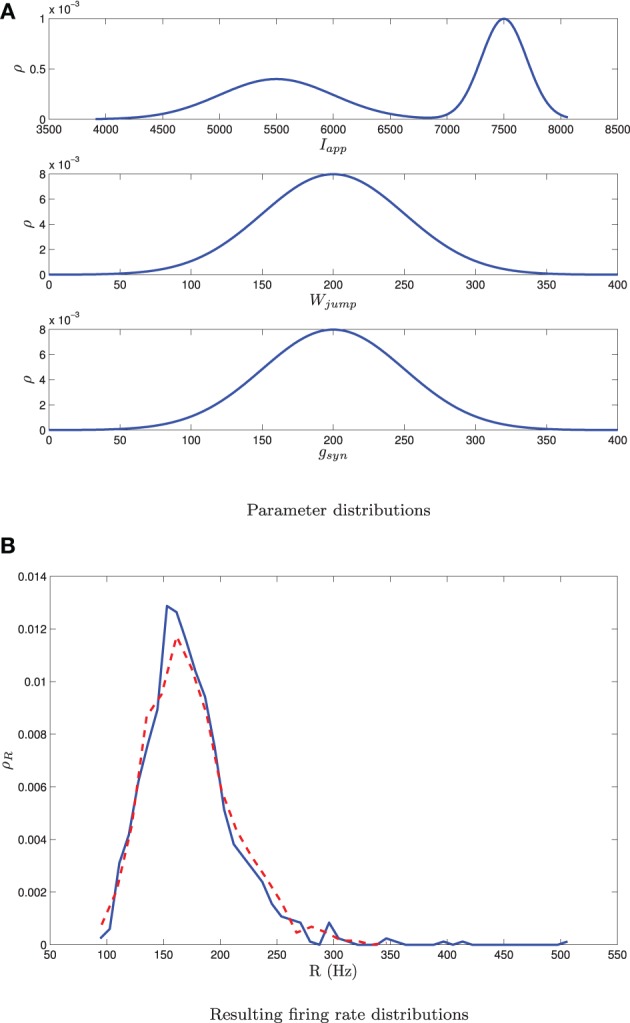
**A bimodal distribution in *I*_app_ together with unimodal distributions in *g*_syn_ and *W*_jump_ as shown in **(A)** yields a unimodal distribution in the firing rate [dashed curve in **(B)**]**. The mean-field equations give a good estimate of this distribution [solid curve in **(B)**]. Simulations are for network of 1000 neurons. parameter given in Table 1 except *I, g*, and *W*_jump_. These are distributed with σ_*w*_ = 50, 〈*W*_jump_〉 = 200, σ_*g*_ = 50, 〈*g*_syn_〉 = 200, *m* = 0.5, 〈*I*_app, 1_〉 = 7500, σ_*I*, 1_ = 200, 〈*I*_app, 2_〉 = 5500, σ_*I*, 2_ = 500. Other combinations of bimodal and unimodal parameter distributions may yield bimodal firing rate distributions. Note that this is different than the situation shown in Figure 9, where a single bimodal source of heterogeneity yielded a bimodal firing rate distribution.

Mean-field III can also be used to determine steady state firing rates following the procedure in section 3.2.2, however, one now has to discretize the equations over a multi-dimensional mesh. While this approach is feasible, we found it is more efficient to predict the steady state firing rates of the individual neurons through the following interpolation scheme. Given knowledge of the parameter distribution, we generate sample points **β**_*i*_ from this distribution. We then generate a steady state firing rate for each sample point, *R*_*i*_ = *g*(**β**_*i*_), where *g* is determined from MFIII as described in section 3.2.2. We interpolate over the (**β**_*i*_, *R*_*i*_) ordered pairs to determine the firing rates of the individual neurons, given knowledge of their parameter values. If we only need the distribution of the firing rates, then the distribution of the *R*_*i*_ is an estimate of this, without need for interpolation. This approach has been applied to a network of 1000 Izhikevich neurons with three simultaneous sources of heterogeneity, as shown in Figure 12.

The one application we found difficult to extend to the case of multiple sources of heterogeneity was the mapping of the distribution of steady state firing rates to the distribution of parameters. There is a fundamental difficulty with this inversion problem: the firing rate distribution is one-dimensional, but the distribution of parameters is multidimensional. Thus, we leave further investigation of this problem for future work.

#### 3.3.1. Bifurcation analysis with multiple sources of heterogeneity—case study

To conclude our work, we consider a realistic model for a CA3 hippocampal network of pyramidal cells. Hemond et al. (2008) classify CA3 pyramidal cells into three types: weakly adapting, strongly adapting and intrinsically bursting. We will focus on the effect on network bursting of having two subpopulations: one strongly adapting and one weakly adapting. We use the Izhikevich model (Equations 4–6) with the parameters set up by Dur-e-Ahmad et al. (2012) (see Table 1), but include heterogeneity in *I*_app_, *g*_syn_ and the adaptation parameters *W*_jump_, τ_*W*_. The parameter distributions are generated through distribution mixing (see Appendix B) of normal distributions with the parameters given in Table 2. We have treated the mean values of *I*_app_ and *g*_syn_ from the strongly adapting subpopulation as the bifurcation parameters. We also varied the proportion of strongly adapting neurons in the population, i.e., parameter *p* in Equation (A8).

**Table 2:** **Table of parameters for the strongly and weakly adapting heterogeneous subpopulations**.

**Parameter**	**Strongly adapting**	**Weakly adapting**
〈*g*_syn_〉	0–600 nS	200 nS
〈σ_*g*_〉	0.5〈*g*_syn_〉	50 nS
〈*I*_app_〉	1000–4000 pA	1200 pA
σ_*I*_	500 pA	500 pA
〈*W*_jump_〉	200 pA	100 pA
σ_*W*_*j*__	50 pA	20 pA
〈τ_*w*_〉	200 ms	50 ms
σ_τ_*w*__	50 ms	10 ms

**Figure 13:**
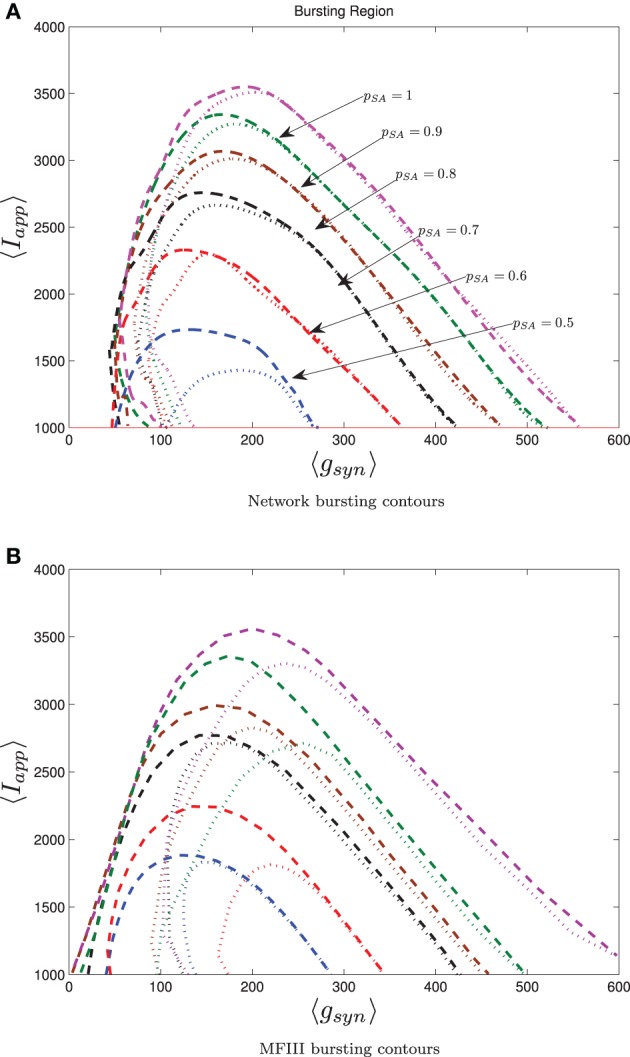
**Case study: the proportion of bursting neurons in a network with both strongly adapting and weakly adapting neurons**. Parameters are as in Table 1 except the *I*_app_, *g*_syn_, *W*_jump_, and τ_*W*_ which have bimodal distributions generated by distribution mixing with parameters given in Table 2. The parameter *p*_*SA*_ represents the proportion of strongly adapting neurons in the network (see Equation A8). The dashed line is the 0% bursting contour, while the dotted line is the 100% bursting contour. As shown in both the network **(A)** and MFIII **(B)**, the bursting regions becomes significantly smaller when the proportion of strongly adapting neurons decreases. In all cases, the curves are smoother spline fits to the actual contours.

The 0 and 100% bursting contours for simulations over the two parameter mesh in the 〈*I*_app_〉, 〈*g*_syn_〉 are shown in Figure 13 for both the full network (Figure 13A) and MFIII (Figure 13B). Numerical bifurcation analysis of MFII (not shown) confirms that the bifurcations are similar to when *I*_app_ was the only source of heterogeneity, in particular, on the left boundary of the bursting region the Hopf bifurcation is supercritical, while on the right it is subcritical.

As shown in Figure 13, when the proportion of strongly adapting neurons is decreased, the bursting region decreases. However, unlike previous results (Nicola and Campbell, 2013, Figure 10), the decrease seems to be more pronounced in the high *g*_syn_ region. This is likely due to having truly heterogeneous distributions of parameters, as opposed to splitting a network into two different homogeneous subpopulations as was done in Nicola and Campbell (2013). In all cases, it appears that heterogeneity shifts the bursting region to higher values of *g*_syn_, outside the range of biologically plausible conductances described in our previous work (Nicola and Campbell, 2013).

## 4. Discussion

Building on the mean-field framework for networks of homogeneous oscillators, we extended the mean-field approach to networks of heterogeneous oscillators. This was accomplished through the derivation of three separate mean-field systems, MFI, MFII, and MFIII, with differing applications and regions of validity. We successfully applied numerical bifurcation analysis to MFI and MFII to aid in the understanding of the different behaviors that heterogeneous networks can display, and how they transition between these different types of behaviors. More importantly, however, we have surpassed the natural limitation of mean-field systems: that they can only provide information about the first moments. With a few additional tools, we used MFIII to derive information about distributions of firing rates, and even parameters, given some basic knowledge.

Other researchers (Hansel and Mato, 2003; Vladimirski et al., 2008; Hermann and Touboul, 2012) have derived firing rate distributions for heterogeneous networks, however, these have been derived under differing assumptions. For example, the heterogeneous mean field systems studied by Hansel and Mato (Equations (5.5–5.7) in Hansel and Mato (2003)) and Hermann and Touboul (Equations (1, 2) in Hermann and Touboul (2012)) have similar integral terms to our MFII, however, they are firing rate models. Our models are current/conductance based models. The difference between these two types of equations arises from which time scale is the fastest, that of the synaptic current, or the firing rate. If the firing rate time scale is assumed to be the fastest, then a differential equation for the synaptic current can be obtained (as in our case). If the time scale of the synaptic current is assumed to be the fastest, then one obtains firing rate equations, as in Hansel and Mato (2003) and Hermann and Touboul (2012). The fact that these two different limits result in different kinds of equations were first highlighted in Dayan and Abbott (2001) (section 7.2). Additionally, no adaptation is contained in the rate models in Hansel and Mato (2003) and Hermann and Touboul (2012). Finally, it is likely that the firing rate models shown in Hansel and Mato (2003) cannot display period doubling bifurcations as they have a similar structure to MFII, which misses out on the more complicated bifurcations of the actual network that MFIII can reproduce, due to its PDE nature. The firing rate models in Hermann and Touboul (2012) have a complicated bifurcation structure as they involve two subpopulations (excitatory and inhibitory) leading to a four dimensional ODE system.

The model of Vladimirski et al. (2008) is formulated in terms of an input–output relation for the synaptic conductance, so has a different structure than ours. It involves a distribution of the synaptic depression variable so has some aspects similar to our MFIII, however, no PDE governing the evolution of this variable is derived.

Dur-e-Ahmad et al. (2012) studied adaptation induced bursting in a network of homogeneous Izhikevich neurons, with parameters determined from experimental data on CA3 pyramidal neurons. They showed that, if the adaptation is strong enough, network bursting occurs in large regions of the parameter space consisting of the synaptic conductance, *g*_syn_, and the applied current, *I*_app_. In Nicola and Campbell (2013) we showed that the transition from tonic firing to bursting involves a saddle-node bifurcation of non-smooth limit cycles, followed by a grazing bifurcation and a subcritical Hopf bifurcation. For fixed *I*_app_ greater than rheobase but sufficiently small, there is one transition from tonic firing to bursting at a low *g*_syn_ value and another from bursting back to tonic firing at a higher *g*_syn_ value. Thus the bursting region is a closed semi-circular region in the *g*_syn_, *I*_app_ parameter space. In Nicola and Campbell (2013) we showed that the size of this bursting region is reduced if the network is split into two homogeneous subnetworks, one strongly adapting and one weakly adapting. Here, we used the tools we developed to investigate how this adaptation induced network bursting is affected by heterogeneity in the parameters. Somewhat surprisingly, we have found that adaptation induced network bursting is not very robust to heterogeneity. This has been confirmed by direct simulations of the full network, bifurcation analysis using MFII and analysis of the proportion of bursting neurons in the network using MFIII. This lack of robustness is caused by two changes to the homogeneous case:

1. The low *g*_syn_ Hopf bifurcation point moves toward higher values, thereby decreasing the size of the bursting region.
2. The low *g*_syn_ Hopf bifurcation switches from subcritical to supercritical. This has two effects:
  - The bifurcation direction changes, eliminating the bursting at conductance values less than the bifurcation value.
  - The initial limit cycles created by the bifurcation are small amplitude oscillations in the firing rate as opposed to full bursts, thus the transition to bursting moves to even higher conductance values.

Further, in networks with both weakly and strongly adapting neurons, heterogeneity caused the high *g*_syn_ Hopf bifurcation value to decrease when the proportion of strongly adapting neurons is reduced.

Let us now put our results in the context of experimental results on the CA3 region. Bursting is often seen in these studies (Andersen et al., 2006, section 5.3.5). When the neurons have their synaptic inputs blocked, however, the majority (≈ 80%) of these pyramidal neurons do not display bursting, but different degrees of spike frequency adaptation (Hemond et al., 2008). Thus, it would seem that adaptation induced network bursting should play a role in the CA3 network. However, the biophysically important part of the parameter region is in the low *g*_syn_ region (Nicola and Campbell, 2013). When this fact is taken in conjunction with our results described above, this would seem to weaken the case that adaptation induced network bursting is the only source of bursting in CA3 networks. Some other mechanism seems necessary.

In their study of hippocampal CA3 pyramidal neurons, Hemond and colleagues note that roughly 20% of pyramidal neurons were intrinsically bursting. That is, the neurons burst without any synaptic input for some input current. It may by possible that a small subpopulation of intrinsically bursting neurons can facilitate bursting in the rest of the network, however, this would depend on the conductance values connecting this particular subpopulation to the rest of the network. This hypothesis can be tested relatively easily using a mean-field approach. All that is required is to fit a two-dimensional adapting model to the intrinsically bursting neurons. This is feasible, as has been previously noted, all the two-dimensional adapting neurons can be turned into intrinsically bursting neurons by simple parameter changes (Izhikevich, 2003). The conductance parameter connecting this subpopulation to the rest is best treated as a bifurcation parameter, with some estimate of the range in which it lies in from physiological data.

While an intrinsically bursting subpopulation is the most promising avenue of study with regards to hippocampal bursting, synaptic depression has also been shown to induce bursting in oscillators that cannot otherwise display this behavior. In a model of the developing chick spinal cord, Vladimirski et al. (2008) found that heterogeneity actually makes the bursting more robust, as opposed to less as we have found. Thus it is possible the synaptic depression induced bursting is more robust to heterogeneity than adaptation induced bursting. However, in this study the heterogeneity was via a uniform distribution in the applied current (as opposed to the Gaussian distributions we consider) and typically 〈*I*_app_〉 was close to rheobase, which could also be factors in their results.

In addition to area CA3 in the hippocampus, adaptation induced bursting has also been suggested as a possible mechanism for the generation of velocity controlled oscillators (VCO's) in the entorhinal cortex by Zilli and Hasselmo (2010). The VCO's burst at frequencies that vary with the velocity of the animal. When a subset of VCO's signals are linearly added to a readout neuron, an interference pattern emerges and a grid cell is formed. Zilli and Hasselmo (2010) use a recursively coupled network of homogeneous Izhikevich neurons with adaptation variables given by *W*_jump_ = 100 and 1/τ_*W*_ = 0.03, parameters values such that adaptation induced bursting can occur. The network acts a single velocity controlled oscillator with the burst frequency varying with the velocity of an animal. This is done by fixing the *g*_syn_ parameter at a specific value and inverting the *F*(*I*) curve, where *F* is the frequency of bursts and *I* is the homogeneous applied current to each neuron. Grid cells can be generated by using multiple networks and linearly adding their output currents to a read-out neuron. This was done under uncorrelated noisy inputs arriving to each neuron. However, Zilli and Hasselmo (2010) state that synchrony in the noise (which can come from the animals velocity signal for example) coming to each VCO network can disrupt grid-cell formation. Here, we have shown that a heterogeneous network of oscillators can still maintain a network level oscillation rate, even if the individual neurons have different behaviors. The network level behaviors are predicted from the mean-field systems. Given the fact that the individual neurons are heterogeneous, any synchronized noise input into the individual neurons should become increasingly desynchronized by the differing responses of the individual neurons. As a network level oscillation exists and the heterogeneity will likely desynchronize any noise coming to the individual oscillators, this is a plausible means of generating velocity controlled oscillators. We leave this particular application of mean-field theory for future work.

In either of these applications, a mean-field system may yield valuable insights as to the mechanisms of bursting and the parameter regions where they occur. By carefully choosing the appropriate bifurcation parameters and accounting for the level of heterogeneity in the neurons in the network, one can determine the bifurcation types and behaviors neurons in these different networks display, in addition to estimates of the different distributions to yield insights about the real cells.

### Conflict of interest statement

The authors declare that the research was conducted in the absence of any commercial or financial relationships that could be construed as a potential conflict of interest.

## Appendices

### Appendix A: computing 〈*v*〉 and 〈*v*|β〉

When the quasi-steady state approximation is applicable, a very convenient method emerges for computing the moments of *v*. In particular, the quasi-steady state approximation not only yields the steady state flux, but it also yields the steady state density:

$$\rho (v)=\frac{J(\langle w\rangle ,s)}{F(v)-\langle w\rangle +gs(e_{r}-v)+I}$$

where *J*(〈*w*〉, *s*) is the steady state flux (〈*R*_*i*_(*t*)〉 for *H*(〈*w*〉, *s*) ≥ 0). Obviously with the density, we can compute the moments of any arbitrary function of *v*. For example, 〈*v*〉 is given by

(A1) $$\langle v\rangle =\int_{V}\frac{J(\langle w\rangle ,s)vdv}{F(v)-\langle w\rangle +gs(e_{r}-v)+I}\text{ if }H(\langle w\rangle ,s)\geq 0$$

However, once we cross the switching manifold, Equation (A1) is no longer valid. An approximation has to be made to compute 〈*v*〉 in this region. In particular, we will assume that 〈*F*(*v*)〉 = *F*(〈*v*〉) and that the dynamics of 〈*v*〉 are fast relative to *s* and 〈*w*〉. If so, then the dynamics of 〈*v*〉 are approximately given by

(A2) $${\langle v\rangle }^{\prime }\approx F(\langle v\rangle )-\langle w\rangle +gs(e_{r}-\langle v\rangle )+I$$

and we can solve the steady state equation for 〈*v*〉

$$F(\langle v\rangle )-\langle w\rangle +gs(e_{r}-\langle v\rangle )+I=0$$

However, we have to be careful here. Based on the assumptions on *F*(*v*) we know that there are only two solutions to this system, and we need to solve for the stable one. For the Izhikevich neuron for example, this is given by

(A3) $$\langle v_{-}\rangle =\frac{\alpha +gs}{2}-\sqrt{-H(\langle w\rangle ,s)}$$

Computation of the integral for the Izhikevich neuron, in conjunction with Equation (A3) yields the following equation for 〈*v*〉 over the entire 〈*w*〉, *s* plane:

(A4) $$\langle v\rangle =\{\begin{array}{ll} \frac{\langle R_{i}(t)\rangle }{2}\log\left(\frac{{\left(v_{\text{peak}}-\frac{\alpha +gs}{2}\right)}^{2}+H(\langle w\rangle ,s)}{{\left(v_{\text{reset}}-\frac{\alpha +gs}{2}\right)}^{2}+H(\langle w\rangle ,s)}\right)+\frac{\alpha +gs}{2} & :H(\langle w\rangle ,s)\geq 0\\ \frac{\alpha +gs}{2}-\sqrt{-H(\langle w\rangle ,s)} & :H(\langle w\rangle ,s)<0 \end{array}$$

The same formulas can actually be used for 〈*v*|**β**〉, with the interpretation that they are now explicit functions of **β** In particular, one can show that under tonic firing, 〈*v*|**β**〉 is given by the same formula as 〈*v*〉 when *H*(〈*w*〉, *s*, **β**) ≥ 0. Additionally, the formula for 〈*v*〉 for *H*(〈*w*〉, *s*, **β**) < 0 is also a satisfactory approximation for 〈*v*|**β**〉. To once again reiterate that while the formula for the mean 〈*v*|**β**〉 under heterogeneity is the same as the formula for 〈*v*〉 under homogeneity, this does not imply that 〈*v*〉 = 〈*v*|**β**〉. In fact, to compute 〈*v*〉, one has to compute the traditional integral for it:

$$\langle v\rangle =\int_{\beta }\langle v|\beta \rangle \rho_{\beta }(\beta )d\beta$$

### Appendix B: computing the proportion of bursting neurons in direct simulations

Performing direct simulations of a network is fairly straight forward, but it is somewhat difficult to use the results of the simulations to automatically classify a given neuron, let alone the entire network, as bursting or tonically firing. We considered various classifiers and opted to use the ratio of the largest to the smallest interspike interval for a neuron when the network has reached steady state. For a bursting neuron, the ratio of its largest interspike interval (which is the interburst interval) to its smallest interspike interval should be large. Thus for the *i*th neuron we define

(A5) $$\lambda_{i}=\frac{\max ISI}{\min ISI}$$

where the max/min is determined after a suitable time period of steady state behavior (either bursting or tonically firing). We set a critical ratio, λ_*c*_, and classify neuron *i* as bursting if λ_*i*_ is higher than this ratio. The critical ratio is typically taken to be 2. This is the single neuron classifier. We use the single neuron classifier to make an estimate for the total proportion of bursting neurons in the network as follows

(A6) $$p_{\text{burst}}=\frac{1}{N}\sum_{i=1}h(\lambda_{i}-\lambda_{c})$$

where *h* is the Heaviside function. We can use *p*_burst_ to classify a network by picking some specific critical value of *p*_burst_ as a threshold for a classifier, however, it is more informative to simply plot the contours of *p*_burst_ for a set of simulations run over a mesh in the bifurcation parameters of interest.

### Appendix C: multiple subpopulations via mixed distributions of heterogeneity

In Nicola and Campbell (2013) we considered multiple subpopulations for heterogeneous networks by simulating discrete homogeneous subpopulations within a network. For example, we simulated two subpopulations with two different sets of parameters corresponding to weakly adapting and strongly adapting neurons as a first attempt at studying inhomogeneous networks. However, a more realistic way of analyzing subpopulations in heterogeneous networks, is through the use of mixed distributions. A mixed random variable *Z* is a function of two or more random variables. For example, if *X* and *Y* are random variables with probability density functions *f*_*X*_ and *f*_*Y*_, respectively, then

(A7) $$Z=\{\begin{array}{ll} X & \text{with probability }p\\ Y & \text{with probability 1}-p \end{array}$$

is a mixed random variable with probability density function

(A8) $$f_{Z}(z)=pf_{X}(z)+(1-p)f_{Y}(z).$$

Now consider a network where the adaptation jump size *w*_jump_ comes from two different subpopulations. In one subpopulation, 〈*w*_jump_〉 is large, and in the other, 〈*w*_jump_〉 is small. We can simulate these two subpopulations within an individual (all-to-all) coupled network using heterogeneity and the density function

(A9) $$f_{w\text{jump}}(w)=p_{SA}f_{SA}(w)+(1-p_{SA})f_{WA}(w).$$

Here *p*_*SA*_ denotes the proportion of strongly adapting neurons, *SA* denotes the strongly adapting subpopulation and *WA* denotes the weakly adapting subpopulation. Note that these densities have higher moments than simply 〈*w*_jump_〉. It is the magnitude of these moments that determine whether or not the density in the heterogeneous parameter we are considering is bimodal, indicative of multiple subpopulations. Using this approach we can analyze how the bimodality affects the steady state distribution properties. We can also use more of the data gained from real networks for the purposes of simulation, as opposed to arbitrarily classifying neurons as strongly adapting or weakly adapting. The parameters of the individual density functions can be approximated, along with *p*, using standard statistical approaches (maximum likelihood estimation, for example). More importantly, however, the same mean-field equations apply to a unimodal or a multimodal distribution of heterogeneity.

### Appendix D: differentiation and numerical inversion of parameter distributions

The procedure in section 3.2.2 requires not only the estimation of 〈*R*_*i*_|β〉, but also the calculation of its inverse and the derivative of this inverse. To calculate 〈*R*_*i*_|β〉 the mean field equations (56–58) are discretized in β and the resulting equations are numerically integrated to steady state, which yields the steady state value of 〈*R*_*i*_|β_*j*_〉 at each mesh point, β_*j*_. The inverse *g*^−1^(*r*) is calculated via numerically inverting the steady state 〈*R*_*i*_|β_*j*_〉 as a function of β_*j*_ as follows. The MATLAB function interp1 is used to interpolate values of β given values of *R* using the steady state (〈*R*_*i*_|β_*j*_〉, β_*j*_) mesh points. The derivative of the inverse is calculated using a finite-difference approximation over the mesh:

$$\frac{dg^{-1}}{dr}{|}_{r=r_{j}}\approx \frac{g^{-1}(r_{j+1})-g^{-1}(r_{j})}{r_{j+1}-r_{j}}$$

This is then used to find ρ_*R*_(*r*) at each mesh point via Equation (61).

To implement the computations in section 3.2.3, Equations (76, 77) are discretized in β and numerically integrating to compute the steady state value of 〈*R*_*i*_|β〉 at each mesh point, 〈*R*_*i*_|β_*j*_〉. This is then used to compute $\frac{d}{d\beta }\langle R|\beta \rangle$ through a first order finite-difference approximation over the discrete mesh:

$$\frac{d\langle R_{i}|\beta \rangle }{d\beta }{|}_{\beta =\beta_{j}}\approx \frac{\langle R_{i}|\beta_{j+1}\rangle -\langle R_{i}|\beta_{j}\rangle }{\beta_{j+1}-\beta_{j}}.$$

These two quantities are then used as approximations of *g*(β) and *g*′(β) in Equation (71) to find ρ_β_(β) at each mesh point.

To estimate the parameter values for individual neurons, we take the discretized steady state firing rate, 〈*R*_*i*_|**β**_*j*_〉, calculated as indicated above. We then invert the functional relationship between 〈*R*_*i*_|**β**_*j*_〉 and β_*j*_, and interpolate the β values of the individual neurons using their firing rate, i.e., we treat 〈*R*_*i*_|**β**_*j*_〉 and β_*j*_ as the (*x*_*j*_, *y*_*j*_) points to be interpolated. This yields an estimate of the parameter values for each individual neuron, unlike in the approach for section 3.2.2 which yielded an estimate of the overall distribution. Note that all that is required for this computation is knowledge of the steady state firing rate for each neuron. We use the griddata and the interp1 functions in MATLAB to perform the interpolation.
